# MyD88 dimerization inhibitor ST2825 targets the aggressiveness of synovial fibroblasts in rheumatoid arthritis patients

**DOI:** 10.1186/s13075-023-03145-0

**Published:** 2023-09-25

**Authors:** Sergio Ramirez-Perez, Rushi Vekariya, Surabhi Gautam, Itzel Viridiana Reyes-Perez, Hicham Drissi, Pallavi Bhattaram

**Affiliations:** 1 Department of Orthopaedics, Emory University School of Medicine, Atlanta, GA 30322 USA; 2 Department of Cell Biology, Emory University School of Medicine, Atlanta, GA 30322 USA; 3 Emory Musculoskeletal Institute, Emory University School of Medicine, Atlanta, GA 30329 USA; 4 Department of Molecular Biology and Genomics, University Center for Health Science, University of Guadalajara, 44340 Guadalajara, Jalisco Mexico; 5 Atlanta VA Medical Center, Decatur, GA 30033 USA

**Keywords:** Rheumatoid arthritis, MyD88, ST2825, Cell cycle, Invasion, Inflammation, Synovial fibroblasts

## Abstract

**Background:**

Dimerization of the myeloid differentiation primary response 88 protein (MyD88) plays a pivotal role in the exacerbated response to innate immunity-dependent signaling in rheumatoid arthritis (RA). ST2825 is a highly specific inhibitor of MyD88 dimerization, previously shown to inhibit the pro-inflammatory gene expression in peripheral blood mononuclear cells from RA patients (RA PBMC). In this study, we elucidated the effect of disrupting MyD88 dimerization by ST2825 on the pathological properties of synovial fibroblasts from RA patients (RA SFs).

**Methods:**

RA SFs were treated with varying concentrations of ST2825 in the presence or absence of bacterial lipopolysaccharides (LPS) to activate innate immunity-dependent TLR signaling. The DNA content of the RA SFs was quantified by imaging cytometry to investigate the effect of ST2825 on different phases of the cell cycle and apoptosis. RNA-seq was used to assess the global response of the RA SF toward ST2825. The invasiveness of RA SFs in Matrigel matrices was measured in organoid cultures. SFs from osteoarthritis (OA SFs) patients and healthy dermal fibroblasts were used as controls.

**Results:**

ST2825 reduced the proliferation of SFs by arresting the cells in the G0/G1 phase of the cell cycle. In support of this finding, transcriptomic analysis by RNA-seq showed that ST2825 may have induced cell cycle arrest by primarily inhibiting the expression of critical cell cycle regulators Cyclin E2 and members of the E2F family transcription factors. Concurrently, ST2825 also downregulated the genes encoding for pain, inflammation, and joint catabolism mediators while upregulating the genes required for the translocation of nuclear proteins into the mitochondria and members of the mitochondrial respiratory complex 1. Finally, we demonstrated that ST2825 inhibited the invasiveness of RA SFs, by showing decreased migration of LPS-treated RA SFs in spheroid cultures.

**Conclusions:**

The pathological properties of the RA SFs, in terms of their aberrant proliferation, increased invasiveness, upregulation of pain and inflammation mediators, and disruption of mitochondrial homeostasis, were attenuated by ST2825 treatment. Taken together with the previously reported anti-inflammatory effects of ST2825 in RA PBMC, this study strongly suggests that targeting MyD88 dimerization could mitigate both systemic and synovial pathologies in a variety of inflammatory arthritic diseases.

**Supplementary Information:**

The online version contains supplementary material available at 10.1186/s13075-023-03145-0.

## Background

Rheumatoid arthritis (RA) is a chronic autoimmune form of joint disease mediated by systemic and local inflammatory mechanisms which ultimately promote pathogenic and destructive processes in the synovium of RA patients [1]. These processes are mediated by the interplay between immune cells and synovial fibroblasts (SFs) in the arthritic synovium [1, 2]. SFs turn into imprinted aggressors that promote and maintain inflammatory and degenerative processes in RA [3, 4]. The aggressiveness of RA SFs includes increased proliferation, aberrant cell cycle progression, reduced apoptosis, and exacerbated production of pro-inflammatory and joint catabolism mediators [5–8]. Together, these cellular and molecular changes in the RA SFs promote joint damage in RA and other chronic autoimmune joint diseases [9–11]. The increased invasive capacity of the RA SFs is another major characteristic of their aggressiveness, which promotes the formation of the hyperplasic and granulated synovial tissue called *pannus* and drives cartilage and bone degeneration [12, 13]. These invasive properties of the RA SFs are regulated by the action of several inflammatory mediators. Collectively, these aggressive pathogenic features that resemble the transformed properties of cancer-associated fibroblasts contribute to the destruction of the joint architecture, altered function, and pain sensitization in RA patients.

Innate immune mechanisms that act locally at the level of joints, and systemically at the level of immune cells, sustain the chronic inflammatory milieu in the RA joints [14]. Toll-like receptors (TLRs) belong to a well-characterized group of innate immune receptors that sense distinct damage-associated molecular patterns (DAMPs), pathogen-associated molecular patterns (PAMPs), and pro-inflammatory mediators in the joint and systemic microenvironments [15]. Various autoantibody immune complexes have been defined as the activators of TLR2 and TLR4 pathways [16, 17], strongly supporting their role in systemic and local inflammation in RA. As a result, several efforts have been made to target upstream and downstream molecules involved in the TLR pathways for RA therapy [14, 18, 19]. Preclinical animal models have evidenced the therapeutic potential of targeting TLR2 and TLR4 [20–22]. However, despite the attempts to successfully achieve amelioration of RA remission, clinical trials targeting TLR4 have only shown moderate effects [23], while others remain in the early phase [24]. The lack of significant results in clinical trials suggests that successful targeting of innate immune pathways may require extensive multipronged inhibitory approaches. Therefore, the inhibition of common adaptor proteins that participate in TLR signaling, rather than the receptors themselves, may be a more suitable approach to observe significant clinical effects.

In this regard, myeloid differentiation primary response 88 (MyD88) is a critical adaptor protein for TLR signaling. Intracellular MyD88 dimerization mediates the recruitment of interleukin receptor-associated kinases 1 and 4 (IRAK1/4) and subsequently led to the activation of intracellular signaling cascades, perpetuating inflammatory signaling in the RA synovium [25]. MyD88 activation downstream of TLR2 and TLR4 signaling has been proposed as a key mechanism promoting RA SF proliferation, invasion, and increased production of inflammatory mediators [26–28]. Importantly, activating mutations in MyD88 was shown to cause severe inflammatory arthritis in humans [29]. Therefore, targeting MyD88 could potentially represent a promising approach to control SF aggressiveness in RA patients.

Our previous study revealed that the MyD88 dimerization inhibitor, ST2825, can suppress systemic inflammation in RA and suggested that it may also likely modulate gene expression in the RA synovium [30]. The ST2825 structure has been widely described, and its mechanism-of-action for specifically targeting MyD88 is mediated by interfering with homo-oligomerization of the BB loop at the MyD88 TIR domain, thereby affecting its dimerization [31, 32]. Thus, in the present study, we performed an unbiased transcriptomic evaluation of the effect of ST2825 in SFs from RA patients, supported by biological function studies. Our results demonstrated that the blockade of MyD88 dimerization by ST2825 causes cell cycle arrest, reduces invasiveness, downregulates the expression of pain and inflammatory mediators, and upregulates the expression of genes that support mitochondrial function in SFs from RA patients. Taken together, these findings strongly suggest that ST2825 could potentially mitigate both local and systemic inflammation in RA patients.

## Methods

### Study design

We aimed to elucidate the effect of disrupting MyD88 dimerization by ST2825 on the pathological properties of SFs from RA patients in an unbiased manner using transcriptomic analysis and biological assays. Our working hypothesis is that the synthetic small molecule ST2825 mitigates the aggressive behavior of RA SFs mediated by their proliferative and antiapoptotic capacity, altered expression of inflammatory mediators, and invasive properties.

### Reagents

LPS from *Escherichia coli* (CAT-L-2880, SIGMA®) and ST2825 inhibitor of MyD88 dimerization (Cat. No. A3840, APExBIO) were used for hDF and SFs stimulation. All reagents were reconstituted according to the manufacturer’s instructions. DAPI (CS1-0127-2 mL, Nexcelom Bioscience) was used for cell cycle assays.

### Cell culture

Experiments on human SFs were performed according to the guidelines and approval of the Emory University Institutional Review Board. OA SFs were prepared from the knee synovium collected from OA patients undergoing total joint replacement as described in our previous publications [33, 34]. RA SFs isolated from the knee synovium of RA patients were collected from cadaveric donors within 48 h of death and were purchased from Articular Engineering, LLC. Dermal fibroblasts from healthy human donors (hDF) were purchased from Lonza. Cells were cultured in DMEM with 10% fetal bovine serum (Corning) and 1% penicillin/streptomycin and utilized between passages 3 and 6. Age and sex of the patients from which the SFs were derived are as described in Fig. S2. Cells were incubated at 37 °C in a humidified 5% CO_2_ atmosphere and 95% humidity.

### Cell cycle and apoptosis analysis

The hDF, OA SFs, and RA SFs were cultured in a 12-well plate after the density adjustment at 1 × 10^5^ cells/mL. The percentages of cells in the G0/G1, S, G2/M phases and apoptotic cells were determined at 24, 48, and 72 h after the incubation with 0, 5, and 10 μM of ST2825. Briefly, cells were permeabilized with 70% ethanol, and their nuclei were stained with DAPI (4 nM) and examined by measuring the amount of DNA per single cell in a multifluorescent channel analysis (blue 377/477) on the Celigo Imaging Cytometer, according to Nexcelom Bioscience protocols (Assay ID: Celigo_02_0001). The percentage of apoptotic cells was determined by gating for intact cells containing less than 2 × amount of DNA calculated at the interface of DAPI integrated intensity on the Celigo Imaging Cytometer (Additional file 1: Fig. S5).

### Invasion assay

Cell invasion assays were carried out on RA SFs 3D spheroids according to a previously published protocol using the Celigo Imaging Cytometer [35]. RA SFs were plated in Nexcelom3D Ultra-low Attachment Round Bottom 96-well Plates at a density of 4000 cells/well in 200 μL of medium and cultured for a period of 4 days, following which the formation of spheroids was visually confirmed using an Olympus CKX53 Microscope (× 10 magnification). On day 4, the Corning® Matrigel® matrix was thawed on ice according to the manufacturer’s protocols. LPS alone at a concentration of 30 ng/mL or LPS containing 10 μM ST2825 were added to the Matrigel and distributed into the 96-well plates containing RA SF spheroids. For unstimulated RA SFs, pure Matrigel was added. Matrigel containing 10 μM ST2825 was added as a control. Automated image acquisition and quantification of the invaded area in μm^2^ were performed on the Celigo Imaging Cytometer at 0, 48, 72, and 96 h after the addition of Matrigel, according to the Nexcelom Bioscience protocols (Assay ID: Celigo_03_0002) [35].

### Bulk RNA sequencing

RA SFs were cultured in 6-well plates after the density of 2.5 × 10^5^ cells/well followed by stimulation with 30 ng/mL of LPS, with or without 10 μM of ST2825 for 24 h. ST2825 was added 45 min prior to LPS stimulation. Unstimulated RA SFs were taken as the control group. Additionally, unstimulated OA SFs were cultured and collected after 24 h and were considered as control cells for unstimulated RA SFs. The total RNA was extracted and purified using Direct-zol RNA MicroPrep (Zymo Research) following the manufacturer’s protocol. RNA quality and quantity were assessed using a 2100 Bioanalyzer (Agilent Technologies). Only samples with an RNA integrity number (RIN) > 8.6 were used for sequencing. Three biological replicates were used per condition. Libraries were generated from 250 ng RNA using TruSeq Stranded Total RNA Sample Prep Kit (Illumina). Sequencing was carried out using the NovaSeq 6000 system (Novogene UC Davis Sequencing Center, Novogene Corporation Inc.). FASTQ files from these samples were uploaded to the DNASTAR Lasergene (version 17.3.0.57) and ArrayStar software for analysis. Paired-end reads were mapped to the GRCh37 human genome assembly. RNA levels were normalized using Log2 RPKM (reads per kilobase of exon model per million mapped sequence) by performing Student’s *t*-test for genes at 95% confidence.

### Differential expression and pathway analysis

Differentially expressed genes (DEGs) were identified using the BioJupies web tool [36]. DEGs with *p*-value < 0.05 and fold change (FC) ≥ 1.5 were used to perform volcano plots, heatmaps, and subsequent pathway analysis. QIAGEN Ingenuity Pathway Analysis (IPA) was used to identify distinct up- and downregulated canonical pathways between different conditions in our study. Data was visualized using GraphPad Prism version 9.4.1, VolcaNoseR, and BioJupies web tools [36, 37]. Protein interaction networks of relevant DEGs were identified and analyzed in STRING version 11.5 [38], the interaction score was set at high confidence = 0.700, and a false discovery rate (FDR) cutoff of 0.05 was used to determine up- or downregulated pathways in the network.

### Gene expression analysis by real-time PCR

The cDNA was synthesized from 1 μg of RNA using qScript™ cDNA SuperMix 5X (QuantaBio). Quantitative real-time polymerase chain reaction (qRT-PCR) was performed using PerfeCTa SYBR® Green FastMix 2X (QuantaBio) on the QuantStudio™ 6 Flex Real-Time PCR System (Applied Biosystems™). For this analysis, at least two biological replicates and 3 technical replicates were used. Fold change (FC) was determined through the 2^−ΔΔCt^ method, and *TBP* mRNA level was used as the reference gene. Primer sequences are detailed in Additional file 1: Table S1.

### Protein assays

Cells were harvested and lysed using Pierce™ IP lysis buffer (Thermo Fisher Scientific) containing 1% Halt™ protease and phosphatase inhibitors (Thermo Fisher Scientific). Equal amounts of protein were electrophoresed on 10% SDS–polyacrylamide gels. The primary antibodies used for the western blot are listed in Additional file 1: Table S2. Bands were visualized using ChemiDoc MP Imaging System (Bio-Rad). For luciferase reporter assays plasmids encoding NF-κB (pNL3.2.NF-kB-RE (Promega) and IL-15 [39], RA SFs were transiently transfected with Viromer Red reagent (Origene). The pSV2bgal plasmid was used as a control for transfection efficiency. Cells were treated with LPS (35 ng/mL), ST2825 (10 μM), or both for 24 h. Luciferase and β-galactosidase activities were assayed using the GloMax Discover System (Promega). Reporter activities were normalized for transfection efficiency and reported as fold change in luciferase activity. Assays were performed as triplicates.

### Statistical analysis

Normality tests were performed to determine the data distribution and appropriate descriptive and inferential statistics. The results were analyzed using the software GraphPad Prism version 9.4.1. The specific statistical analysis is described in the corresponding figure caption. One-way ANOVA, two-way ANOVA and Dunnett’s multiple comparisons, Student’s *t*-test, and one-way ANOVA and Tukey’s multiple comparisons test were used. A *p*-value less than 0.05 was considered statistically significant.

## Results

### Effect of inhibiting MyD88 dimerization on hDF and OA SFs

**Fig. 1:**
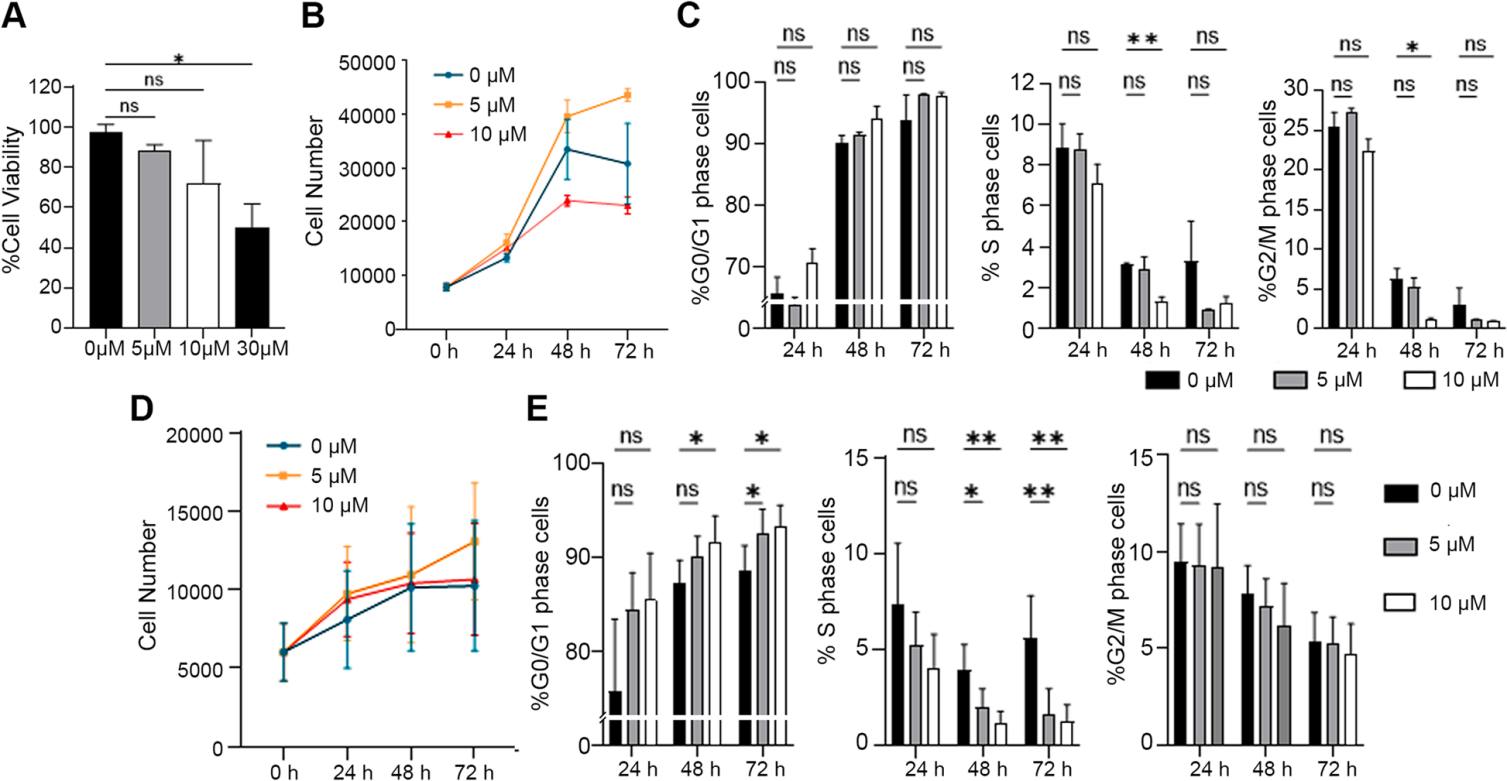
ST2825 prevents S-phase progression in human dermal fibroblasts (hDF) and OA SFs. Effect of ST2825 on hDF (**A**, **B**, **C**) and OA-SFs (**D**, **E**). **A** DAPI-based cell viability assay identified the total number of cells among different conditions in hDF treated with 0, 5, 10, and 30 μM of ST2825 for 24 h. **B** Changes in cell number of hDF at 24, 48, and 72 h after the incubation with 5 and 10 μM of ST2825. **C** Quantification of the percentage of cells in various phases of the cell cycle upon ST2825 treatment at 24, 48, and 72 h by imaging cytometry. **D** Increase in cell number of OA SF at 24, 48, and 72 h after the incubation with 5 and 10 μM of ST2825. **E** Increase in cell number of OA SF at 24, 48, and 72 h after the incubation with 5 and 10 μM of ST2825. *N* = 3; two-way ANOVA and Dunnett’s multiple comparisons test were used to determine statistical significance. *p < 0.05, **p < 0.01

To identify the effects of inhibiting MyD88 dimerization, the viability of hDF incubated with 0, 5, 10, and 30 μM of ST2825 for 24 h was determined (Fig. 1A). The results indicated that 5 and 10 μM of ST2825 did not compromise cell viability; therefore, those two concentrations were used for subsequent experiments. To evaluate the long-term effect of ST2825 on proliferation, hDF and OA SFs were monitored for 24, 48, and 72 h after incubation with 5 and 10 μM of ST2825 (Fig. 1B, D). The analysis reported no statistically significant differences among conditions, suggesting that cell viability was not compromised. Although not significant, we observed a drop in cell number, particularly in hDF treated with 10 μM of ST2825. Thus, the DNA content of hDF and OA SFs was quantified to investigate the effect of ST2825 on different phases of the cell cycle (Fig. 1C, E and Additional file 1: Fig. S1). Our analysis revealed that 10 μM of ST2825 induced a significant decrease in the percentage of phase S cells in hDF and OA SFs, with a corresponding increase in the percentage of G0/G1 phase OA SFs at 48 h. Interestingly, these changes were accentuated in OA SFs treated with both 5 and 10 μM of ST2825 at 72 h, suggesting that ST2825 may influence cell proliferation through induction of G0/G1 cell cycle arrest.

### Transcriptomic analysis to confirm the inflammatory status of the RA SFs used in this study

Since our goal was to determine whether the MyD88 dimerization inhibition mediated by ST2825 modulates pathogenic processes in RA SFs, we confirmed the inflammatory status of the RA SFs samples used in this study by comparing them with OA SFs isolated from OA synovium of patients undergoing total knee replacement (Additional file 1: Fig. S2A). We identified a total of 1879 differentially expressed genes (DEGs) by 1.5-fold change (*p* < 0.05) between RA and OA SFs. The analysis revealed 826 downregulated and 1053 upregulated genes (Additional file 1: Fig. S2B). Hierarchical cluster analysis of normalized gene expression from RA and OA SFs shows 2129 genes at 95% confidence that clustered together in the appropriate group (Additional file 1: Fig. S2C). Upregulated and downregulated genes were further analyzed to identify canonical pathways underlying pathogenic features in RA SFs (Additional file 1: Fig. S2D). The overexpression of important inflammatory genes, along with the increased expression of *MYD88* observed in RA SFs, established the suitability of the RA SFs samples for this study (Additional file 1: Fig. S2E). Importantly, it supports our hypothesis for targeting this protein in SF as a potential pharmacological approach in the arthritic joints.

### ST2825 arrests cell cycle progression in RA SFs

**Fig. 2:**
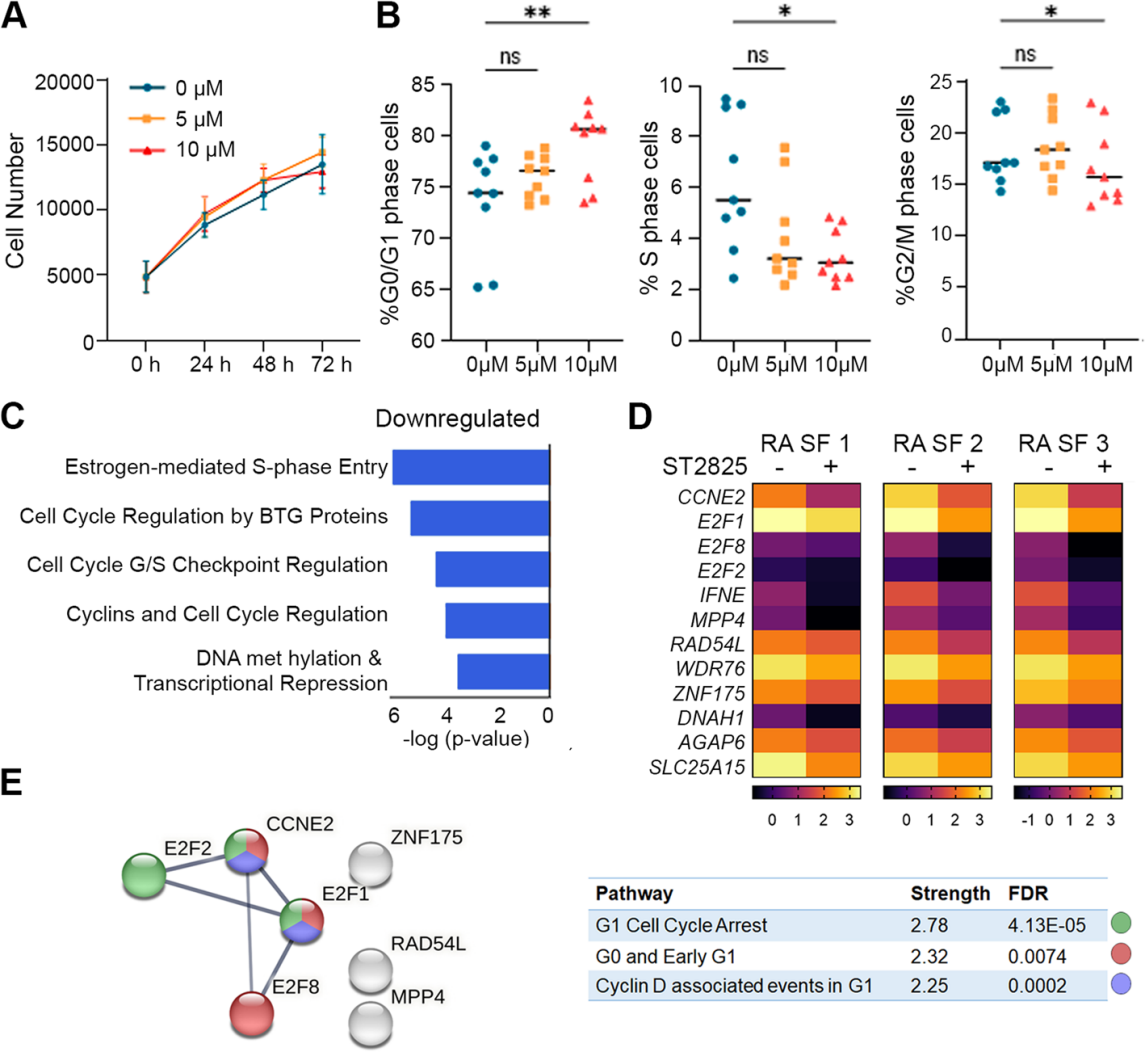
ST2825 arrests the cell cycle progression in RA SFs. **A** Changes in cell numbers at 24, 48, and 72 h upon incubation with 5 and 10 μM of ST2825. **B** Quantification of the percentage of RA SFs in various phases of the cell cycle upon ST2825 treatment at 24 h by imaging cytometry. **A**, **B** One-way ANOVA and Dunnett’s multiple comparisons test were performed to determine statistical significance. **C** Ingenuity Pathway Analysis (Qiagen) showing the top 5 canonical pathways predicted to be associated with the genes downregulated in RA SFs. **D** Heatmaps of Log2 RPKM values of genes associated with downregulated canonical pathways. **E** Interatomic gene cluster by STRING showing the association of the downregulated genes with cell cycle progression. Association strength and false discovery rate (FDR) *p*-values are presented

To determine the biological effects of ST2825 on RA SFs, we monitored cell proliferation for 24, 48, and 72 h after incubation with 5 and 10 μM of ST2825 (Fig. 2A). Although cell numbers remained unchanged, the treatment with 10 μM of ST2825 caused a significant increase in the percentage of G0/G1 phase and significantly decreased the percentage of S and G2/M phase RA SFs (Fig. 2B). The same analysis was performed on RA SFs incubated with ST2825 for 48 and 72 h, even though a similar trend was observed, the changes in cell cycle phases were not significant suggesting that the ST2825 acted within 24 h of the treatment (Additional file 1: Figs. S3 and S4). We, therefore, selected the 24 h time point for transcriptomic analysis by RNA-seq. Genes were selected based on the reactome pathway enrichment (Additional file 1: Fig. S6 and Additional file 2: Supplementary Data 1), and the main downregulated pathways were related to the cell cycle, which matches with the results obtained for the analysis of the cell cycle states. Those downregulated genes with 1.5 FC and *p* < 0.05 were further analyzed through QIAGEN Ingenuity Pathway Analysis (IPA), and common genes across downregulated pathways were selected for heatmaps. The transcriptomic analysis revealed cell cycle regulation among the top five IPA canonical pathways downregulated by ST2825 (Fig. 2C). We next performed a paired differential gene expression analysis in the 3 independent RA SFs samples; heatmaps show that ST2825 treatment successfully downregulated cell cycle-related genes (Fig. 2D). The predicted protein interaction network showed the main gene cluster involved in the regulation of G1 cell cycle arrest (green), G0 and early G1 (red), and Cyclin D-associated events in G1 (blue) at high interaction confidence = 0.700 and PPI enrichment *p*-value = 1.43E − 07 (Fig. 2E). The percentage of apoptotic cells was evaluated in all time points among conditions; significant differences in apoptosis were only observed at 72 h with 10 μM of ST2825 (Additional file 1: Fig. S5). Taken together, these observations suggest that MyD88 dimerization inhibition arrests cell cycle progression by inducing G0/G1 cell cycle arrest, which may potentially modulate the exacerbated proliferation and apoptosis resistance observed in RA SFs.

### ST2825 inhibits the pathological gene expression signature of LPS-stimulated RA SFs

**Fig. 3:**
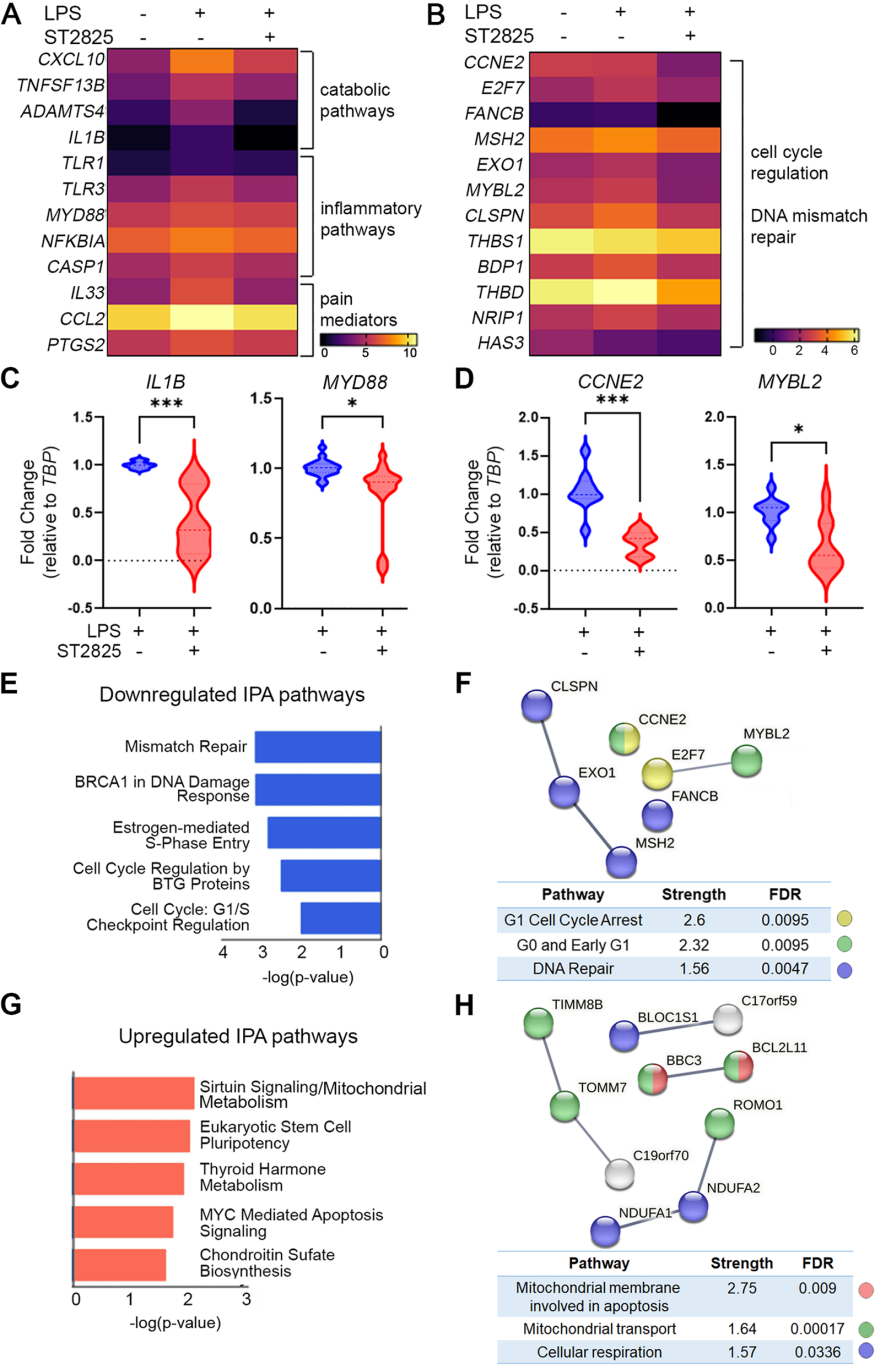
ST2825 inhibits the pathological gene expression of LPS-stimulated RA SFs. RA SFs were stimulated with LPS to evaluate the effect of ST2825 on gene expression molecules involved in inflammation and cell cycle. **A** Heatmap shows the overall expression of inflammatory genes in RA SFs stimulated with LPS and LPS plus ST2825. Three biological replicates were used per each condition. Values are shown in RPKM Log2. **C** Downregulated catabolic genes in RA SFs treated with LPS plus ST2825 were confirmed by qRT-PCR. Two biological replicates were analyzed in triplicates to calculate FC by the 2^−ΔΔCt^ method, and statistical significance was calculated by Student’s *t*-test. **B** Heatmap shows the overall expression of cell cycle-related genes in RA SFs stimulated with LPS and LPS plus ST2825. Three biological replicates were used per each condition. Values are shown in RPKM Log2. **D** Downregulated cell cycle-related genes in RA SFs treated with LPS plus ST2825 were confirmed by qRT-PCR. Two biological replicates were analyzed in triplicates to calculate FC by the 2^−ΔΔCt^ method, and statistical significance was calculated by Student’s *t*-test. **E** Transcriptomic analysis identified the top five downregulated canonical pathways in RA SFs treated with LPS plus ST2825. **F** Interatomic gene cluster shows the main downregulated molecules controlling cell cycle progression in RA SFs treated with LPS plus ST2825. Transcriptomic analysis identified the top five upregulated canonical pathways in RA SFs treated with LPS plus ST2825. **H** Interatomic gene cluster shows the main upregulated molecules controlling mitochondrial processes in RA SFs treated with LPS plus ST2825. *p < 0.05, ***p < 0.001

We next investigated the effect of ST2825 under inflammatory conditions by stimulating RA SFs with LPS, a well-known pro-inflammatory inductor known to activate TLR signaling upstream of MyD88 dimerization. RNA sequencing was performed from LPS-stimulated RA SFs after 24 h (Additional file 1: Fig. S7 and Additional file 3: Supplementary Data 2). In order to investigate the possible role of ST2825 in modulating the expression of critical genes involved with RA SF aggressiveness, we manually selected some genes associated with catabolic and inflammatory pathways along with pain mediators (Fig. 3A). The analysis revealed upregulation of critical genes involved in catabolic processes such as *CXCL10*, *TNFSF13B*, *ADAMTS4* (ADAM metallopeptidase with thrombospondin type 1 motif 4), and *IL1B* (Interleukin 1 beta). Similarly, the upregulation of several genes related to inflammatory pathways such as *TLR1* (Toll-like receptor 1), *TLR3* (Toll-like receptor 3), *CASP1*, and *MYD88* was observed. It is important to mention that some of these genes were also upregulated in RA SF vs. OA SF in Additional file 1: Fig. S2, supporting our hypothesis that ST2825 can target distinct aspects of RA SF aggressiveness. One of the most interesting findings was the LPS-mediated upregulation of interleukin 33 (*IL33*), C–C motif chemokine ligand 2 (*CCL2*), and prostaglandin-endoperoxide synthase 2 (*PTGS2*), genes that have been described as pain mediators (Fig. 3A). Differential gene expression analysis also showed that the most downregulated genes by the effect ST2825 on LPS-stimulated RA SFs were those related to cell cycle regulation and DNA mismatch repair (Fig. 3B, E). We successfully validated the ST2825-induced downregulation of the pro-inflammatory genes *IL1B* and *MYD88* and the cell cycle regulators *CCNE2* and *MYBL2* (Myb-related protein B) genes by qRT-PCR (Fig. 3C, D). The predicted STRING protein interaction network showed that the main gene cluster involved in the regulation of G1 cell cycle arrest (yellow), G0 and early G1 (green), and DNA repair (blue) at high interaction confidence = 0.700 and PPI enrichment *p*-value = 0.00431 (Fig. 3F). Upregulated genes by the effect ST2825 on LPS-stimulated RA SFs were related to Sirtuin signaling, mitochondrial metabolism, pluripotency, apoptosis, and chondroitin sulfate biosynthesis (Fig. 3G). The predicted STRING protein interaction network also showed that upregulated genes participate in distinct mitochondrial processes; the analysis was performed at high interaction confidence = 0.700, and the PPI enrichment *p*-value was 4.25E − 08 (Fig. 3H).

### ST2825 suppresses the invasive features of LPS-stimulated RA SFs in 3D spheroid cultures

**Fig. 4:**
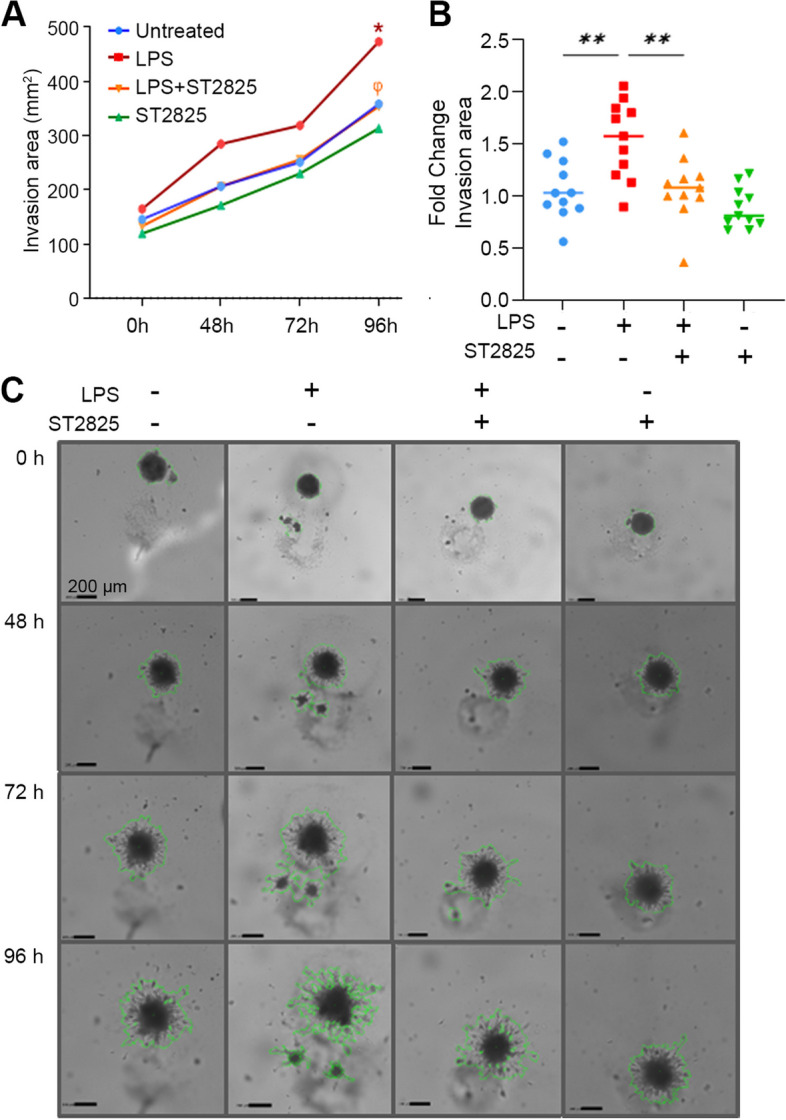
ST2825 suppresses the invasiveness of LPS-stimulated RA SFs 3D spheroids. RA SFs 3D spheroids were performed and stimulated with LPS to potentiate their invasive capacity on the Matrigel matrix. **A** To monitor the long-term effect of ST2825 suppressing invasiveness, spheroids were evaluated at 48, 72, and 96 h. Three biological replicates with at least three technical replicates per sample were used. The line graph shows the invaded area in mm^2^. Mixed-effects model restricted maximum likelihood (REML) and Tukey’s multiple comparisons were implemented for the statistical analysis. *p < 0.01, untreated vs. LPS (96 h); *φ p* < 0.01, LPS vs. LPS + ST2825 (96 h). **B** The fold change for the area of invasion was calculated for individual values considering untreated cells as the control group. Significant suppression of invasiveness in LPS-stimulated RA SFs 3D spheroids treated with ST2825 at 96 h was observed. One-way ANOVA and Tukey’s multiple comparisons test were performed. **p < 0.01. **C** Representative image shows the differences in invasion area quantification (scale bar = 200 μm) among conditions at 0, 48, 72, and 96 h

Invasiveness represents a key pathogenic feature of RA SFs due to their contribution to *pannus* formation and joint destruction. In this study, LPS was used to potentiate the invasive properties of RA SFs. In order to evaluate long-term changes, spheroids were performed and monitored for 48, 72, and 96 h after incubation with LPS or LPS plus ST2825 (Fig. 4A). The area of the invasion reached statistical significance at 96 h, an effect that was significantly suppressed by the MyD88 inhibitor ST2825 (Fig. 4B, C). Taken together, the data obtained from this analysis demonstrates that local treatment with ST2825 may suppress the invasiveness of RA SFs contributing to ameliorating joint damage.

### ST2825 targets NF-κB-dependent mechanisms and IKK-related kinases in RA SFs

**Fig. 5:**
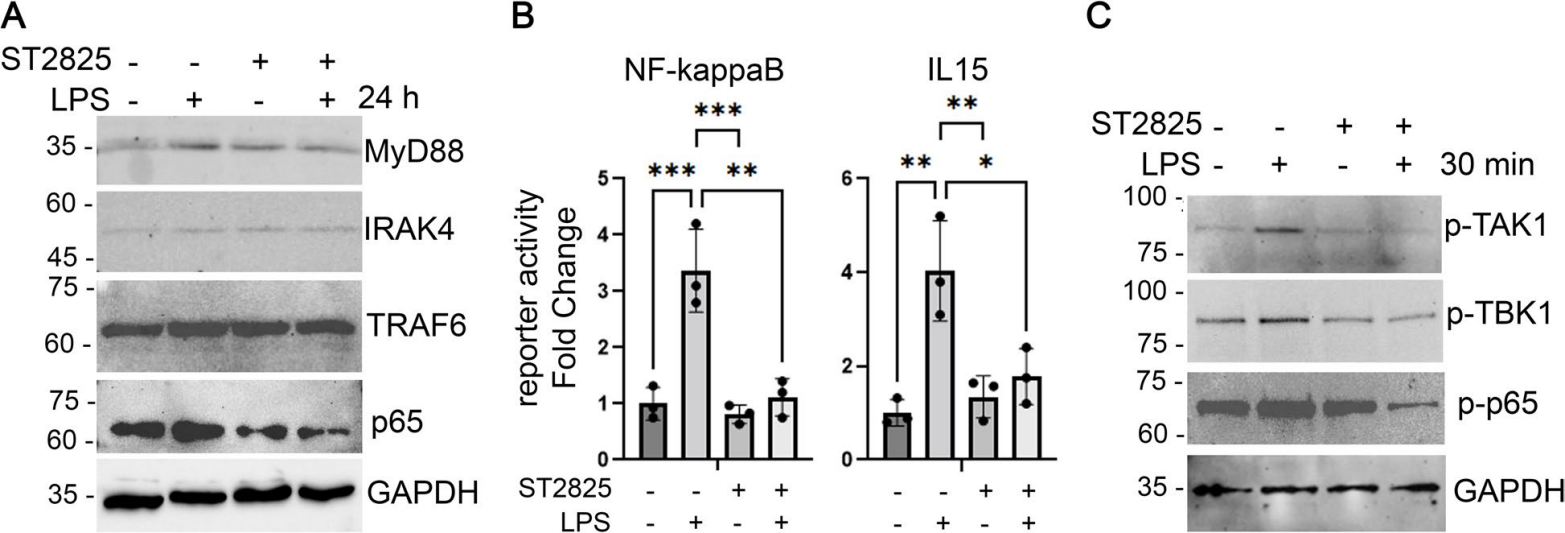
ST2825 targets RA SF aggressiveness by suppressing NF-κB-dependent mechanisms and IKK-related kinases. RA SFs were stimulated with LPS to evaluate the effect of ST2825 on protein expression molecules involved in the Myddosome formation and the NF-κB signaling. **A** Protein expression is shown for MyD88, IRAK4, TRAF6, and p65 after 24 h of stimulation with LPS (35 ng/mL), ST2825 (10 μM), or both. **B** Luciferase activity was determined in two NF-κB-binding site reporters (NF-κB and IL15) after 24 h of treatment with LPS (35 ng/mL), ST2825 (10 μM), or both. Three independent experiments were performed to calculate the statistical significance. One-way ANOVA and Tukey’s multiple comparisons test were performed. *p < 0.05, **p < 0.01, ***p < 0.001. **C** Activation of the IKK-related kinases (TAK1 and BTK1) and NF-κB p65 was tested after 30 min of treatment with LPS (70 ng/mL), ST2825 (10 μM), or a combination of both. GAPDH protein expression was used as an internal control

In order to elucidate the effect of ST2825 on MyD88 and NF-κB signaling pathways in RA SFs, we treated cells with LPS and ST2825 and performed western blot and luciferase reporter assays. Our results show that the levels of MyD88, IRAK4, and TRAF6 were not significantly changed by ST2825 (Fig. 5A and Additional file 1: Fig. S8). However, this result was expected as ST2825 inhibits MyD88 dimerization and subsequent signaling events by disrupting the formation of the Myddosome complex but does not directly affect the gene expression or protein level of MyD88 or its interacting partners. Nonetheless, we were able to demonstrate that the level of p65, which is the downstream effector of MyD88, was downregulated by ST2825 (Fig. 5A). In addition, our results showed that targeting MyD88 by ST2825 significantly inhibits p65-dependent NF-κB binding site reporter luciferase activity (Fig. 5B) and may have an additional effect by inactivating the IKK-related kinases TAK1 and TBK1, and NF-κB p65 in RA SFs treated with LPS (Fig. 5C and Additional file 1: Fig. S8).

## Discussion

**Fig. 6:**
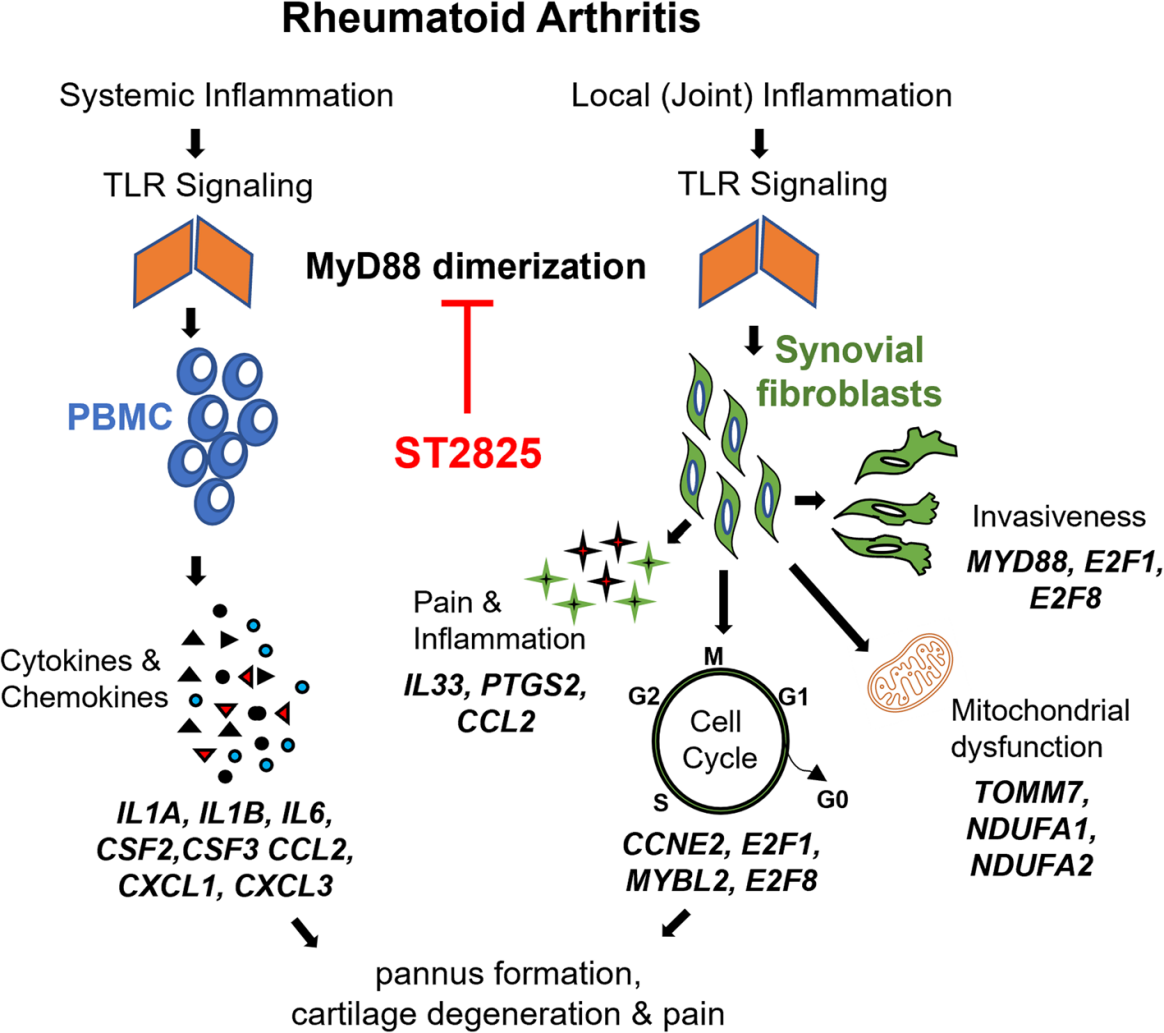
The hypothesis of MyD88-dependent systemic and local pathogenic mechanisms in RA. MyD88 dimerization drives systemic and local inflammatory and destructive processes. ST2825 effectively targets systemic inflammation in RA PBMC and local RA SFs aggressiveness by inhibiting cell cycle progression, invasion, and production of inflammatory and pain mediators, promoting apoptosis, and likely restoring mitochondrial homeostasis

ST2825 is a halogenated heptapeptide that competitively binds to the Toll interleukin-1 receptor (TIR) domain of MyD88 and blocks its dimerization, a critical event in the TLR/IL-1 signaling pathway, which culminates in the activation of NF-κB signaling [31]. We previously showed that ST2825 inhibited the overexpression and release of chemokines and cytokines on PBMC [30, 40]. In this study, we show that MyD88 inhibition by ST2825 effectively reduced the aggressiveness of RA SFs via arresting cell cycle progression, inhibiting the expression of pain and inflammatory mediators, decreasing their invasiveness, and promoting mitochondrial function. Based on these findings from our previous and current studies performed in RA PBMC and RA SFs, respectively, we hypothesize that ST2825 could potentially mitigate the chronic activation of both systemic and local inflammatory processes in RA patients (Fig. 6). Our data also show that ST2825 inhibited cell cycle progression in OA SFs and hDF, suggesting that it could be developed as a therapeutic for OA and psoriatic arthritis.

One of the major findings of this study is that ST2825 decreased cell proliferation of RA SFs by arresting the cells in the G0/G1 phase of the cell cycle as early as 24 h after treatment. This arrest in the cell cycle may have contributed to the increased number of apoptotic nuclei at 72 h after treatment [41]. Moreover, pro-apoptotic genes BCL2 like 1 (*BCL2L1*, *also known as BCLXL*) and BCL2-binding component 3 (*BBC3*, also known as *PUMA*) were upregulated by ST2825 in LPS-stimulated RA SFs, suggesting that ST2825 may relieve the apoptotic resistance of RA SFs. Several studies have reported that MyD88 is linked to cell proliferation. *MYD88* activating mutations were shown to result in the aberrant proliferation of multiple types of cancer cells [42–45]. In particular, MyD88 was reported to play a role in the occurrence and development of breast cancer [46], epithelial ovarian carcinoma [47], human hepatocellular carcinoma [48], osteosarcoma [49], adenocarcinoma of the colon [50], and hematopoietic cancer-like lymphoma and leukemia [45]. ST2825 was reported to inhibit the growth and apoptosis resistance of lymphoid malignancies, but the underlying molecular mechanism is incompletely understood [45]. Our findings show that ST2825 may play an inhibitory role in cell cycle progression by primarily downregulating the expression of critical cell cycle checkpoint regulators such as Cyclin E2 (*CCNE2*) and the members of the E2F family transcription factors (*E2F1*, *E2F2*, *E2F7*, and *E2F8*). CCNE2 is an important component in cell proliferation [51] and cancer [52], which may explain the anti-proliferative activity of ST2825. Regarding the E2F transcription factor family, previous reports have revealed its crucial regulatory role in the timely expression of genes needed for cell cycle progression and proliferation [53]. RA synovial tissue exhibits much greater levels of *E2F2* expression. E2F2 has also been linked to RA pathology by potentiating the aggressive characteristics of RA SFs, including proliferation, invasion, and in vitro cytokine production [54]. Moreover, E2F2 binds to the *MYD88* promoter and upregulates its expression, which subsequently activates the PI3K/AKT/NF-κB pathway [27].

The current knowledge suggests that abrogating MyD88 functions will most likely mitigate cell proliferation. According to the increased percentage of cells in phase G0/G1 and decreased percentage in S and G2/M phases found in our study, along with downregulated genes associated with cell cycle progression; the treatment with 10 μM of ST2825 causes a cell cycle arrest in G1. Therefore, cell proliferation can be prevented. These results match with several reports which have demonstrated that TLR4/MyD88 blockade can suppress the cell proliferation of gastric cancer cell lines [55], hepatocarcinoma HEPG2 cells [56], human hepatoma cell lines [43], and head kidney lymphocytes in an infectious model [57]. In RA, abrogating the TLR4/MyD88/NF-κB axis has demonstrated beneficial effects in SFs [58–60]. Due to the tumor-like phenotype displayed by RA SFs, we speculate that a similar mechanism is displayed in the arthritic joint.

In the present study, invasiveness was successfully targeted by ST2825 on LPS-stimulated RA SF spheroids. In this regard, previous studies have reported suppression of LPS-induced migration and invasion of breast cancer cells after targeting MyD88-dependent signaling pathways, for instance, MyD88/GSK-3β/Snail and MyD88/NF-κB/Snail [44]. Osteosarcoma cell migration was also abolished after treatment with ST2825 suggesting that MyD88 plays a major role in increasing the proliferation rate and reducing apoptosis [49]. Increased expression of *E2F1* in RA SFs has been strongly associated with pro-proliferative and pro-invasive effects through activation of the TGFβ-activated MAPK signaling. E2F1 seems to participate in modulating the expression of proangiogenic factors, vascular endothelial growth factor (*VEGF*) and hypoxia-inducible factor 1 subunit alpha (*HIF1A*), and cell cycle progression genes (*CCNE2* and *MYBL2*), which are critical for synovial hyperplasia in RA [61]. ST2825 has been previously reported as a repressor of *MYBL2* expression, which is a central regulator of cell proliferation, cell survival, and differentiation in tumorigenesis [62]. Our transcriptomic analysis has revealed that ST2825 downregulates those critical genes involved in migration, which suggests that the downstream effect of ST2825 will most likely result in suppressing the expression of cell cycle and proliferation-dependent genes on LPS-stimulated RA SFs.

Expression of several pro-inflammatory genes in LPS-stimulated RA SFs was downregulated after ST2825 treatment. The overexpression of *IL1B*, a key inflammatory mediator involved in RA pathogenesis, was significantly decreased by ST2825. Beyond the MyD88 dimerization inhibition, ST2825 significantly decreased the expression of *MYD88*. This effect might be attributed to the decreased level of its transcriptional activator E2F2, suggesting an additional regulatory mechanism for inflammation in RA orchestrated by ST2825 [27]. The anti-inflammatory effects of ST2825 described in neuroinflammation suggest that this inhibitor is capable of suppressing pro-inflammatory mediators and oxidative stress by inhibiting the ROS/NLRP3/Caspase-1 signaling pathway [63]. Taken together, these and our previous findings observed in RA PBMC, we speculated that ST2825 anti-inflammatory effects are able to prevent both local and systemic inflammation in RA patients.

Pain is a clinically relevant consequence of chronic inflammation in RA patients. In this regard, preclinical studies performed in animal models of RA and OA have shown that the blockade of IL-33 has the potential to reduce both pain and joint damage [64, 65]. At the mechanistic level, IL-33 may increase pain sensitivity via upregulating cyclooxygenase 2 (*COX2*) expression [66, 67]. CCL2-CCR2 interaction and downstream signaling were reported to excite sensory neurons and contribute to knee hyperalgesia in an experimental OA mouse model [68–70]. Our data show that ST2825 downregulates *IL33*, *PTGS2* (encodes for COX2), and *CCL2* expression in RA SFs. Thus, ST2825 may have an added therapeutic advantage as a pain medication for RA patients.

Studies in the BV2 neuronal cell line showed that ST2825 downregulated *COX2* expression and decreased the production of reactive oxygen species [63]. Our data suggest that ST2825 not only acts by downregulating *COX2* expression but also exerts its mito-protective effects by upregulating genes encoding for mitochondrial proteins like TOMM7, TIMM8B, and NADH:ubiquinone oxidoreductase subunits, NDUFA1 and NDUFA2. TOMM7 is a mitochondrial translocase that plays an essential role in the translocation of nuclear-encoded mitochondrial proteins into mitochondria and regulates the coupling between oxygen consumption and ATP synthesis [71, 72]. Mutations in *TOMM7* have resulted in syndromic short stature in humans [71, 73]. NDUFA1 and NDUFA2 are components of the mitochondrial respiratory complex I, which utilizes the NADH produced by Krebs Cycle to play a central role in energy metabolism [71, 74]. Interestingly, an expression quantitative trait locus (eQTL) study identified phosphorylation-associated single nucleotide polymorphisms (SNPs) linked with the *NDUFA2* gene as significant RA risk alleles [75]. Although the role of these mitochondrial proteins in RA SF pathology is not well understood, based on reports from other cell and tissue types, we speculate that their upregulation by ST2825 may counteract the mitochondrial dysfunction and restore energy homeostasis in RA SFs.

This study also elucidated the potential effect of ST2825 on targeting NF-κB-dependent mechanisms in RA SFs. Our results indicated that disrupting the Myddosome formation decreased the level of p65 and its activation. In addition, the activation of the IKK-related kinases, TAK1 and TBK1, was decreased by the effect of ST2825. Supporting our results, a previous study performed in bone marrow-derived macrophages (BMDMs) reported that LPS stimulation was able to induce the activation of TBK1 via the MyD88-dependent pathway, while in MyD88^−/−^ BMDMs the activation was reduced [76]. Additionally, TAK1 can initiate the activation of the IKK canonical complex by TLRs in a MyD88-dependent pathway in distinct cell types [76–78]. TAK1 inhibition has also been associated with decreased cell proliferation and cytokine secretion in malignant B cells [78]. Taken together, these findings suggest that ST2825 mechanism-of-action could be dependent on inhibition of the IKK-related kinases, TBK1 and TAK1, and subsequent suppression of IKK canonical kinases (IKKα/β) converging on NF-κB p65 inactivation.

Our study did not consider the disease stage or disease activity score, which are important limitations. Additionally, the inclusion of treatments used for RA and OA patients may have revealed additional findings in SFs. Experiments with *MYD88* siRNA could have provided proof for our speculation regarding the vital role of MyD88 driving the aggressive behavior of RA SFs; however, those experiments were not technically feasible due to the low transfection efficiency of *MYD88* siRNA in RA SFs.

## Conclusions

In summary, our results show that ST2825 inhibits multiple aspects of the RA SF aggressive features by arresting their cell cycle progression, inhibiting the production of inflammation and pain mediators, decreasing invasion, promoting apoptosis, and likely restoring mitochondrial homeostasis. Although this study is limited to in vitro experiments in a small sample size, our results provide a robust and unbiased justification to undertake future studies to evaluate the effects of ST2825 in a greater number of RA patient-derived SFs. Furthermore, our comprehensive RNA-seq analysis will serve as a resource to understand the importance of MyD88 dimerization in other inflammatory diseases. In the future, preclinical studies in animal models must be coupled with single-cell RNA sequencing to decipher the safety and efficacy of ST2825 on various subtypes of RA SFs and immune cell populations.

### Supplementary Information

**Additional file 1: Fig. S1.** Cell cycle analysis on hDF and OA SFs treated with ST2825. Representative histograms show cell cycle differences among different ST2825 concentrations and time points. G0/G1-phase cells, S-phase cells, and G2/M-phase cells are shown in the figure for hDF (A) and OA SFs (B). The y-axes represent the total count of cells, and the x-axes represent integrated intensity of DAPI measured by whole-well image cytometry. **Fig. S2.** Transcriptomic analysis revealed the upregulation of critical inflammatory mediators in RA SFs. (A) Description of OA and RA SFs used in RNA-seq experiments. (B) Volcano plot of up and downregulated genes in RA SFs compared OA SFs by 1.5 FC and *p*-value < 0.05 (C) Hierarchical clustering of the genes differentially expressed genes by Euclidean distance and centroid linkage method (D). Ingenuity Pathway Analysis (Qiagen) showing the top 5 canonical pathways predicted to be associated with the genes up and downregulated in RA SFs. (E) Heatmaps Log2 RPKM values of genes associated with canonical pathways identified in panel D. **Fig. S3.** Cell cycle analysis of RA-FLS treated with ST2825 at 48 and 72 h. Quantification of the percentage of RA SFs in various phases of the cell cycle upon ST2825 treatment at 48 (A) and 72 (B) h by imaging cytometry. One-way ANOVA and Dunnett’s multiple comparisons test were performed to determine statistical significance. *p<0.05, ****p<0.0001. **Fig. S4.** Cell cycle analysis on RA SFs treated with ST2825. Representative histograms show cell cycle differences among different ST2825 concentrations and time points. Apoptotic cells (yellow), G0/G1-phase cells (green), S-phase cells (blue), and G2/Mphase cells (purple) are shown in the figure. The y axes represent the total count of cells, and the x axes represents integrated intensity of DAPI measured by whole-well image cytometry. **Fig. S5.** Apoptosis analysis of RA-FLS treated with ST2825. Apoptosis was determined on RA SFs treated with 0, 5, and 10 μM of ST2825 at 0, 24, 48, and 72 h. No statistically significant differences were observed after 24 and 48 h of incubation with ST2825. The percentage of apoptotic cells significantly increased after 72 h of incubation with 10 μM of ST2825 (*p*=0.0476). Two-way ANOVA and Dunnett’s multiple comparisons test were used to determine statistical significane. *p<0.05. **Fig. S6.** Transcriptomic analysis identified DEGs and canonical pathways in RA SFs treated with ST2825. (A) Volcano plot of up and downregulated genes in RA SFs compared ST2825-treated RA SFs by 1.5 FC and *p*-value < 0.05. Enriched pathway analysis showing the top 10 canonical downregulated (B) and upregulated (C) pathways predicted to be associated with the genes up and downregulated in RA SFs treated with ST2825. **Fig. S7.** Transcriptomic analysis identified the effect of ST2825 on DEGs and canonical pathways in LPS-treated RA SFs. (A) Volcano plot of up and downregulated genes in LPS-stimulated RA SFs after treatment with ST2825 by 1.5 FC and *p*-value < 0.05. Enriched pathway analysis showing the top 10 canonical downregulated (B) and upregulated (C) pathways predicted to be associated with the genes up and downregulated in RA SFs. **Fig S8.** Raw data of western blot images. (A) Fig 5A and (B) Fig 5C. **Table S1.** Primer sequences for qRT-PCR. **Table S2.** Antibodies used for western blot.

**Additional file 2: Supplementary Data 1.** Reactome Pathway Enrichment.

**Additional file 3: Supplementary Data 2.** Reactome Pathway Enrichment LPS-stimulated RA.

## Data Availability

All data underlying the findings of this article are fully available without restriction. RNA-seq data is deposited at the international public repository of Gene Expression Omnibus (GEO/NCBI) database as the study number GSE217012.

## Abbreviations

RA: Rheumatoid arthritis
OA: Osteoarthritis
SFs: Synovial fibroblasts
PBMC: Peripheral blood mononuclear cells
MyD88: Myeloid differentiation primary response 88
E2F: E2F family transcription factors
CCNE2: Cyclin E2
PAMPs: Pathogen-associated molecular patterns
DAMPs: Damage-associated molecular patterns
LPS: Lipopolysaccharides
TLR: Toll-like receptors
TNF: Tumor necrosis factor
*CXCL10*: C-X-C motif chemokine ligand 10
*TLR3*: Toll-like receptor 3
*TNFSF13B*: TNF superfamily member 13b
*CASP1*: Caspase 1
*IL1B*: Interleukin 1
*IL33*: Interleukin 33
*ADAMTS4*: ADAM metallopeptidase with thrombospondin type 1 motif 4
*IL1B*: Interleukin 1 beta
*TLR1*: Toll-like receptor 1
*TLR3*: Toll-like receptor 3
*CCL2*: C-C motif chemokine ligand 2
*PTGS2*: Prostaglandin-endoperoxide synthase 2
IRAK1/4: Interleukin receptor-associated kinases 1 and 4
NF-κB: Nuclear factor kappa light chain enhancer of activated B cells
BCL: B cell lymphoma 2
*BCL2L1*: BCL2 like 1
*BBC3*: BCL2-binding component 3
NADH: Nicotinamide adenine dinucleotide (reduced form)
eQTL: Expression quantitative trait locus
SNPs: Single nucleotide polymorphisms
TIR: Toll interleukin-1 receptor
hDF: Human dermal fibroblasts
RIN: RNA integrity number
DEGs: Differentially expressed genes
CLEAR: Coordinated lysosomal expression and regulation
*VEGF*: Vascular endothelial growth factors
*HIF1A*: Hypoxia-inducible factor 1 subunit alpha
*MYBL2*: Myb-related protein B
IKK: Inhibitor of nuclear factor kappa-B (IκB) kinase complex
TBK1: TANK-binding kinase 1
TAK1: Transforming growth factor-β (TGF-β)-activated kinase 1

## References

[1] Komatsu N, Takayanagi H (2022). Mechanisms of joint destruction in rheumatoid arthritis - immune cell-fibroblast-bone interactions. Nat Rev Rheumatol.

[2] Tu J, Huang W, Zhang W, Mei J, Zhu C (2022). Two main cellular components in rheumatoid arthritis: communication between T cells and fibroblast-like synoviocytes in the joint synovium. Front Immunol.

[3] Bottini N, Firestein GS (2013). Duality of fibroblast-like synoviocytes in RA: passive responders and imprinted aggressors. Nat Rev Rheumatol.

[4] Nygaard G, Firestein GS (2020). Restoring synovial homeostasis in rheumatoid arthritis by targeting fibroblast-like synoviocytes. Nat Rev Rheumatol.

[5] Woods JM, Klosowska K, Spoden DJ, Stumbo NG, Paige DJ, Scatizzi JC (2006). A cell-cycle independent role for p21 in regulating synovial fibroblast migration in rheumatoid arthritis. Arthritis Res Ther.

[6] Xu N, Wang Y, Li D, Chen G, Sun R, Zhu R (2010). MDM4 overexpression contributes to synoviocyte proliferation in patients with rheumatoid arthritis. Biochem Biophys Res Commun.

[7] Taranto E, Xue JR, Lacey D, Hutchinson P, Smith M, Morand EF (2005). Detection of the p53 regulator murine double-minute protein 2 in rheumatoid arthritis. J Rheumatol.

[8] Hu Z, Chen Y, Zhu S, Feng X, Zhang B, Huang J (2022). Sonic Hedgehog promotes proliferation and migration of fibroblast-like synoviocytes in rheumatoid arthritis via Rho/ROCK signaling. J Immunol Res.

[9] Mizoguchi F, Slowikowski K, Wei K, Marshall JL, Rao DA, Chang SK (2018). Functionally distinct disease-associated fibroblast subsets in rheumatoid arthritis. Nat Commun.

[10] Zhang F, Wei K, Slowikowski K, Fonseka CY, Rao DA, Kelly S (2019). Defining inflammatory cell states in rheumatoid arthritis joint synovial tissues by integrating single-cell transcriptomics and mass cytometry. Nat Immunol.

[11] Korsunsky I, Wei K, Pohin M, Kim EY, Barone F, Major T (2022). Cross-tissue, single-cell stromal atlas identifies shared pathological fibroblast phenotypes in four chronic inflammatory diseases. Med (N Y).

[12] Tolboom TC, van der Helm-Van Mil AH, Nelissen RG, Breedveld FC, Toes RE, Huizinga TW (2005). Invasiveness of fibroblast-like synoviocytes is an individual patient characteristic associated with the rate of joint destruction in patients with rheumatoid arthritis. Arthritis Rheum.

[13] Laragione T, Brenner M, Sherry B, Gulko PS (2011). CXCL10 and its receptor CXCR3 regulate synovial fibroblast invasion in rheumatoid arthritis. Arthritis Rheum.

[14] Joosten LA, Abdollahi-Roodsaz S, Dinarello CA, O’Neill L, Netea MG (2016). Toll-like receptors and chronic inflammation in rheumatic diseases: new developments. Nat Rev Rheumatol.

[15] Elshabrawy HA, Essani AE, Szekanecz Z, Fox DA, Shahrara S (2017). TLRs, future potential therapeutic targets for RA. Autoimmun Rev.

[16] Sohn DH, Rhodes C, Onuma K, Zhao X, Sharpe O, Gazitt T (2015). Local Joint inflammation and histone citrullination in a murine model of the transition from preclinical autoimmunity to inflammatory arthritis. Arthritis Rheumatol.

[17] Manivel VA, Sohrabian A, Rönnelid J (2016). Granulocyte-augmented chemokine production induced by type II collagen containing immune complexes is mediated via TLR4 in rheumatoid arthritis patients. Eur J Immunol.

[18] Lee JH, Cho ML, Kim JI, Moon YM, Oh HJ, Kim GT (2009). Interleukin 17 (IL-17) increases the expression of Toll-like receptor-2, 4, and 9 by increasing IL-1beta and IL-6 production in autoimmune arthritis. J Rheumatol.

[19] Achek A, Yesudhas D, Choi S (2016). Toll-like receptors: promising therapeutic targets for inflammatory diseases. Arch Pharm Res.

[20] Joosten LA, Koenders MI, Smeets RL, Heuvelmans-Jacobs M, Helsen MM, Takeda K (2003). Toll-like receptor 2 pathway drives streptococcal cell wall-induced joint inflammation: critical role of myeloid differentiation factor 88. J Immunol.

[21] Abdollahi-Roodsaz S, Joosten LA, Roelofs MF, Radstake TR, Matera G, Popa C (2007). Inhibition of Toll-like receptor 4 breaks the inflammatory loop in autoimmune destructive arthritis. Arthritis Rheum.

[22] Achek A, Shah M, Seo JY, Kwon HK, Gui X, Shin HJ (2019). Linear and rationally designed stapled peptides abrogate TLR4 pathway and relieve inflammatory symptoms in rheumatoid arthritis rat model. J Med Chem.

[23] Monnet E, Choy EH, McInnes I, Kobakhidze T, de Graaf K, Jacqmin P (2020). Efficacy and safety of NI-0101, an anti-toll-like receptor 4 monoclonal antibody, in patients with rheumatoid arthritis after inadequate response to methotrexate: a phase II study. Ann Rheum Dis.

[24] Hernández-Jiménez M, Martín-Vílchez S, Ochoa D, Mejía-Abril G, Román M, Camargo-Mamani P (2022). First-in-human phase I clinical trial of a TLR4-binding DNA aptamer, ApTOLL: safety and pharmacokinetics in healthy volunteers. Mol Ther Nucleic Acids.

[25] Sacre SM, Andreakos E, Kiriakidis S, Amjadi P, Lundberg A, Giddins G (2007). The Toll-like receptor adaptor proteins MyD88 and Mal/TIRAP contribute to the inflammatory and destructive processes in a human model of rheumatoid arthritis. Am J Pathol.

[26] Zhan H, Chen H, Tang Z, Liu S, Xie K, Wang H (2022). SIX1 attenuates inflammation and rheumatoid arthritis by silencing MyD88-dependent TLR1/2 signaling. Int Immunopharmacol.

[27] Wang S, Wang L, Wu C, Sun S, Pan JH (2018). E2F2 directly regulates the STAT1 and PI3K/AKT/NF-κB pathways to exacerbate the inflammatory phenotype in rheumatoid arthritis synovial fibroblasts and mouse embryonic fibroblasts. Arthritis Res Ther.

[28] Cho ML, Ju JH, Kim HR, Oh HJ, Kang CM, Jhun JY (2007). Toll-like receptor 2 ligand mediates the upregulation of angiogenic factor, vascular endothelial growth factor and interleukin-8/CXCL8 in human rheumatoid synovial fibroblasts. Immunol Lett.

[29] Sikora KA, Bennett JR, Vyncke L, Deng Z, Tsai WL, Pauwels E (2018). Germline gain-of-function myeloid differentiation primary response gene-88 (MYD88) mutation in a child with severe arthritis. J Allergy Clin Immunol.

[30] Ramirez-Perez S, Oregon-Romero E, Reyes-Perez IV, Bhattaram P (2021). Targeting MyD88 downregulates inflammatory mediators and pathogenic processes in PBMC from DMARDs-naïve rheumatoid arthritis patients. Front Pharmacol.

[31] Loiarro M, Capolunghi F, Fantò N, Gallo G, Campo S, Arseni B (2007). Pivotal advance: inhibition of MyD88 dimerization and recruitment of IRAK1 and IRAK4 by a novel peptidomimetic compound. J Leukoc Biol.

[32] Loiarro M, Ruggiero V, Sette C (2013). Targeting the Toll-like receptor/interleukin 1 receptor pathway in human diseases: rational design of MyD88 inhibitors. Clin Lymphoma Myeloma Leuk.

[33] Jones K, Angelozzi M, Gangishetti U, Haseeb A, de Charleroy C, Lefebvre V (2021). Human adult fibroblast-like synoviocytes and articular chondrocytes exhibit prominent overlap in their transcriptomic signatures. ACR Open Rheumatol.

[34] Bhattaram P, Muschler G, Wixler V, Lefebvre V (2018). Inflammatory cytokines stabilize SOXC transcription factors to mediate the transformation of fibroblast-like synoviocytes in arthritic disease. Arthritis Rheumatol.

[35] Vinci M, Box C, Eccles SA (2015). Three-dimensional (3D) tumor spheroid invasion assay. J Vis Exp.

[36] Torre D, Lachmann A, Ma’ayan A (2018). BioJupies: automated generation of interactive notebooks for RNA-Seq data analysis in the cloud. Cell Syst.

[37] Goedhart J, Luijsterburg MS (2020). VolcaNoseR is a web app for creating, exploring, labeling and sharing volcano plots. Sci Rep.

[38] Szklarczyk D, Gable AL, Lyon D, Junge A, Wyder S, Huerta-Cepas J (2019). STRING v11: protein-protein association networks with increased coverage, supporting functional discovery in genome-wide experimental datasets. Nucleic Acids Res.

[39] Jones K, Ramirez-Perez S, Niu S, Gangishetti U, Drissi H, Bhattaram P (2022). SOX4 and RELA function as transcriptional partners to regulate the expression of TNF-responsive genes in fibroblast-like synoviocytes. Front Immunol.

[40] Ramírez-Pérez S, Hernández-Palma LA, Oregon-Romero E, Anaya-Macías BU, García-Arellano S, González-Estevez G (2020). Downregulation of inflammatory cytokine release from IL-1β and LPS-stimulated PBMC orchestrated by ST2825, a MyD88 dimerisation inhibitor. Molecules.

[41] Pucci B, Kasten M, Giordano A (2000). Cell cycle and apoptosis. Neoplasia.

[42] Zhu G, Cheng Z, Huang Y, Zheng W, Yang S, Lin C (2020). MyD88 mediates colorectal cancer cell proliferation, migration and invasion via NF-κB/AP-1 signaling pathway. Int J Mol Med.

[43] Xu X, Yin Y, Tang J, Xie Y, Han Z, Zhang X (2017). Long non-coding RNA Myd88 promotes growth and metastasis in hepatocellular carcinoma via regulating Myd88 expression through H3K27 modification. Cell Death Dis.

[44] Liu JH, Chen C, Li ZY, Zou ZM, Gao DC, Zhang X (2020). The MyD88 inhibitor TJ-M2010-2 suppresses proliferation, migration and invasion of breast cancer cells by regulating MyD88/GSK-3β and MyD88/NF-κB signalling pathways. Exp Cell Res.

[45] Shiratori E, Itoh M, Tohda S (2017). MYD88 inhibitor ST2825 suppresses the growth of lymphoma and leukaemia cells. Anticancer Res.

[46] Xiang F, Ni Z, Zhan Y, Kong Q, Xu J, Jiang J (2016). Increased expression of MyD88 and association with paclitaxel resistance in breast cancer. Tumour Biol.

[47] Zhu Y, Huang JM, Zhang GN, Zha X, Deng BF (2012). Prognostic significance of MyD88 expression by human epithelial ovarian carcinoma cells. J Transl Med.

[48] Liang B, Chen R, Wang T, Cao L, Liu Y, Yin F (2013). Myeloid differentiation factor 88 promotes growth and metastasis of human hepatocellular carcinoma. Clin Cancer Res.

[49] Chen J, He J, Yang Y, Jiang J (2018). An analysis of the expression and function of myeloid differentiation factor 88 in human osteosarcoma. Oncol Lett.

[50] Salcedo R, Worschech A, Cardone M, Jones Y, Gyulai Z, Dai RM (2010). MyD88-mediated signaling prevents development of adenocarcinomas of the colon: role of interleukin 18. J Exp Med.

[51] Fukuda K, Miura Y, Maeda T, Hayashi S, Kuroda R (2019). Expression profiling of genes in rheumatoid fibroblast-like synoviocytes regulated by tumor necrosis factor-like ligand 1A using cDNA microarray analysis. Biomed Rep.

[52] Lee C, Fernandez KJ, Alexandrou S, Sergio CM, Deng N, Rogers S (2020). Cyclin E2 promotes whole genome doubling in breast cancer. Cancers (Basel).

[53] Ren B, Cam H, Takahashi Y, Volkert T, Terragni J, Young RA (2002). E2F integrates cell cycle progression with DNA repair, replication, and G(2)/M checkpoints. Genes Dev.

[54] Zhang R, Wang L, Pan JH, Han J (2018). A critical role of E2F transcription factor 2 in proinflammatory cytokines-dependent proliferation and invasiveness of fibroblast-like synoviocytes in rheumatoid Arthritis. Sci Rep.

[55] Yue Y, Zhou T, Gao Y, Zhang Z, Li L, Liu L (2017). High mobility group box 1/toll-like receptor 4/myeloid differentiation factor 88 signaling promotes progression of gastric cancer. Tumour Biol.

[56] Liu Y, Li T, Xu Y, Xu E, Zhou M, Wang B (2016). Effects of TLR4 gene silencing on the proliferation and apotosis of hepatocarcinoma HEPG2 cells. Oncol Lett.

[57] Zhou Y, Chen X, Cao Z, Li J, Long H, Wu Y (2020). R848 is involved in the antibacterial immune response of golden pompano (Trachinotus ovatus) through TLR7/8-MyD88-NF-κB-signaling pathway. Front Immunol.

[58] Li Y, Xu JZ, Gu CX, Liu GL, Tian K (2019). Carvacrol suppresses inflammatory responses in rheumatoid arthritis fibroblast-like synoviocytes. J Cell Biochem.

[59] Yao RB, Zhao ZM, Zhao LJ, Cai H (2017). Sinomenine inhibits the inflammatory responses of human fibroblast-like synoviocytes via the TLR4/MyD88/NF-κB signaling pathway in rheumatoid arthritis. Pharmazie.

[60] Wu R, Long L, Chen Q, Wu X, Zhu J, Zhou B (2017). Effects of Tim-3 silencing on the viability of fibroblast-like synoviocytes and lipopolysaccharide-induced inflammatory reactions. Exp Ther Med.

[61] Ainsworth RI, Hammaker D, Nygaard G, Ansalone C, Machado C, Zhang K (2022). Systems-biology analysis of rheumatoid arthritis fibroblast-like synoviocytes implicates cell line-specific transcription factor function. Nat Commun.

[62] Musa J, Aynaud MM, Mirabeau O, Delattre O, Grünewald TG (2017). MYBL2 (B-Myb): a central regulator of cell proliferation, cell survival and differentiation involved in tumorigenesis. Cell Death Dis.

[63] Zhang SS, Liu M, Liu DN, Shang YF, Wang YH, Du GH (2022). ST2825, a small molecule inhibitor of MyD88, suppresses NF-κB activation and the ROS/NLRP3/cleaved caspase-1 signaling pathway to attenuate lipopolysaccharide-stimulated neuroinflammation. Molecules.

[64] He Z, Song Y, Yi Y, Qiu F, Wang J, Li J (2020). Blockade of IL-33 signalling attenuates osteoarthritis. Clin Transl Immunology.

[65] Li Y, Fu Y, Chen H, Liu X, Li M (2020). Blocking interleukin-33 alleviates the joint inflammation and inhibits the development of collagen-induced arthritis in mice. J Immunol Res.

[66] Li Y, Shi J, Qi S, Zhang J, Peng D, Chen Z (2018). IL-33 facilitates proliferation of colorectal cancer dependent on COX2/PGE(2). J Exp Clin Cancer Res.

[67] Chen L, Song Z, Cao X, Fan M, Zhou Y, Zhang G (2022). Interleukin-33 regulates the endoplasmic reticulum stress of human myometrium via an influx of calcium during initiation of labor. Elife.

[68] Ishihara S, Obeidat AM, Wokosin DL, Ren D, Miller RJ, Malfait AM (2021). The role of intra-articular neuronal CCR2 receptors in knee joint pain associated with experimental osteoarthritis in mice. Arthritis Res Ther.

[69] Miller RE, Tran PB, Das R, Ghoreishi-Haack N, Ren D, Miller RJ (2012). CCR2 chemokine receptor signaling mediates pain in experimental osteoarthritis. Proc Natl Acad Sci U S A.

[70] Miotla Zarebska J, Chanalaris A, Driscoll C, Burleigh A, Miller RE, Malfait AM (2017). CCL2 and CCR2 regulate pain-related behaviour and early gene expression in post-traumatic murine osteoarthritis but contribute little to chondropathy. Osteoarthritis Cartilage.

[71] Young C, Batkovskyte D, Kitamura M, Shvedova M, Mihara Y, Akiba J (2023). A hypomorphic variant in the translocase of the outer mitochondrial membrane complex subunit TOMM7 causes short stature and developmental delay. HGG Adv.

[72] Hasson SA, Kane LA, Yamano K, Huang CH, Sliter DA, Buehler E (2013). High-content genome-wide RNAi screens identify regulators of parkin upstream of mitophagy. Nature.

[73] Garg A, Keng WT, Chen Z, Sathe AA, Xing C, Kailasam PD (2022). Autosomal recessive progeroid syndrome due to homozygosity for a TOMM7 variant. J Clin Invest.

[74] Huttula S, Väyrynen H, Helisalmi S, Kytövuori L, Luukkainen L, Hiltunen M (2022). NDUFA1 p.Gly32Arg variant in early-onset dementia. Neurobiol Aging.

[75] He P, Jiang F, Guo W, Guo YF, Lei SF, Deng FY (2021). PhosSNPs-regulated gene network and pathway significant for rheumatoid arthritis. Hum Hered.

[76] Clark K, Takeuchi O, Akira S, Cohen P (2011). The TRAF-associated protein TANK facilitates cross-talk within the IkappaB kinase family during Toll-like receptor signaling. Proc Natl Acad Sci U S A.

[77] He A, Ji R, Shao J, He C, Jin M, Xu Y (2016). TLR4-MyD88-TRAF6-TAK1 complex-mediated NF-κB activation contribute to the anti-inflammatory effect of V8 in LPS-induced human cervical cancer SiHa cells. Inflammation.

[78] Ansell SM, Hodge LS, Secreto FJ, Manske M, Braggio E, Price-Troska T (2014). Activation of TAK1 by MYD88 L265P drives malignant B-cell growth in non-Hodgkin lymphoma. Blood Cancer J.

