# Supplementary material for: MyD88 dimerization inhibitor ST2825 targets the aggressiveness of synovial fibroblasts in rheumatoid arthritis patients
Source: Arthritis Res Ther. 2023 Sep 25;25:180. doi: 10.1186/s13075-023-03145-0 (PMC10519089; doi:10.1186/s13075-023-03145-0)
Supplement: Supplementary file 1 — Additional file 1: Fig. S1. Cell cycle analysis on hDF and OA SFs treated with ST2825. Representative histograms show cell cycle differences among different ST2825 concentrations and time points. G0/G1-phase cells, S-phase cells, and G2/M-phase cells are shown in the figure for hDF (A) and OA SFs (B). The y-axes represent the total count of cells, and the x-axes represent integrated intensity of DAPI measured by whole-well image cytometry. Fig. S2. Transcriptomic analysis revealed the upregulation of critical inflammatory mediators in RA SFs. (A) Description of OA and RA SFs used in RNA-seq experiments. (B) Volcano plot of up and downregulated genes in RA SFs compared OA SFs by 1.5 FC and p-value < 0.05 (C) Hierarchical clustering of the genes differentially expressed genes by Euclidean distance and centroid linkage method (D). Ingenuity Pathway Analysis (Qiagen) showing the top 5 canonical pathways predicted to be associated with the genes up and downregulated in RA SFs. (E) Heatmaps Log2 RPKM values of genes associated with canonical pathways identified in panel D. Fig. S3. Cell cycle analysis of RA-FLS treated with ST2825 at 48 and 72 h. Quantification of the percentage of RA SFs in various phases of the cell cycle upon ST2825 treatment at 48 (A) and 72 (B) h by imaging cytometry. One-way ANOVA and Dunnett’s multiple comparisons test were performed to determine statistical significance. *p<0.05, ****p<0.0001. Fig. S4. Cell cycle analysis on RA SFs treated with ST2825. Representative histograms show cell cycle differences among different ST2825 concentrations and time points. Apoptotic cells (yellow), G0/G1-phase cells (green), S-phase cells (blue), and G2/Mphase cells (purple) are shown in the figure. The y axes represent the total count of cells, and the x axes represents integrated intensity of DAPI measured by whole-well image cytometry. Fig. S5. Apoptosis analysis of RA-FLS treated with ST2825. Apoptosis was determined on RA SFs treated with 0, 5, and 10 μM o [file 13075_2023_3145_MOESM1_ESM.pdf]

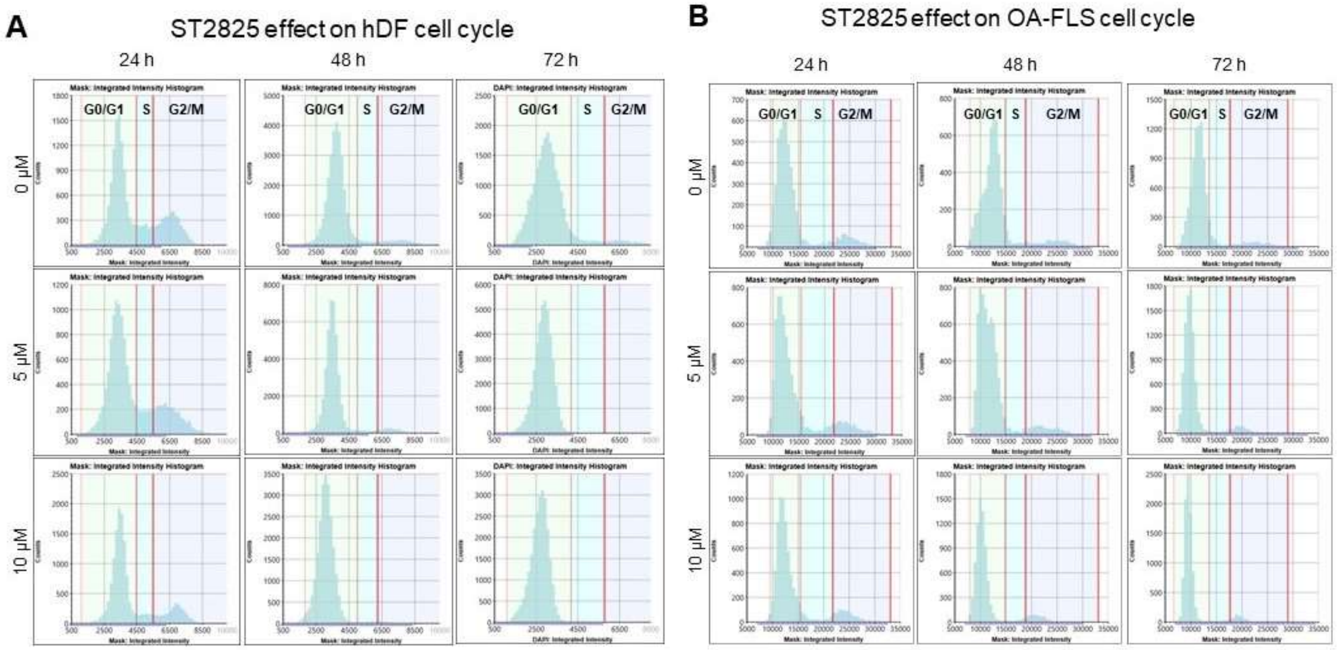

Fig. S1. Cell cycle analysis on hDF and OA SFs treated with ST2825.

Representative histograms show cell cycle differences among different ST2825 concentrations and time points. G0/G1-phase cells, S-phase cells, and G2/M-phase cells are shown in the figure for hDF (A) and OA SFs (B). The y-axes represent the total count of cells, and the x-axes represent integrated intensity of DAPI measured by whole-well image cytometry.

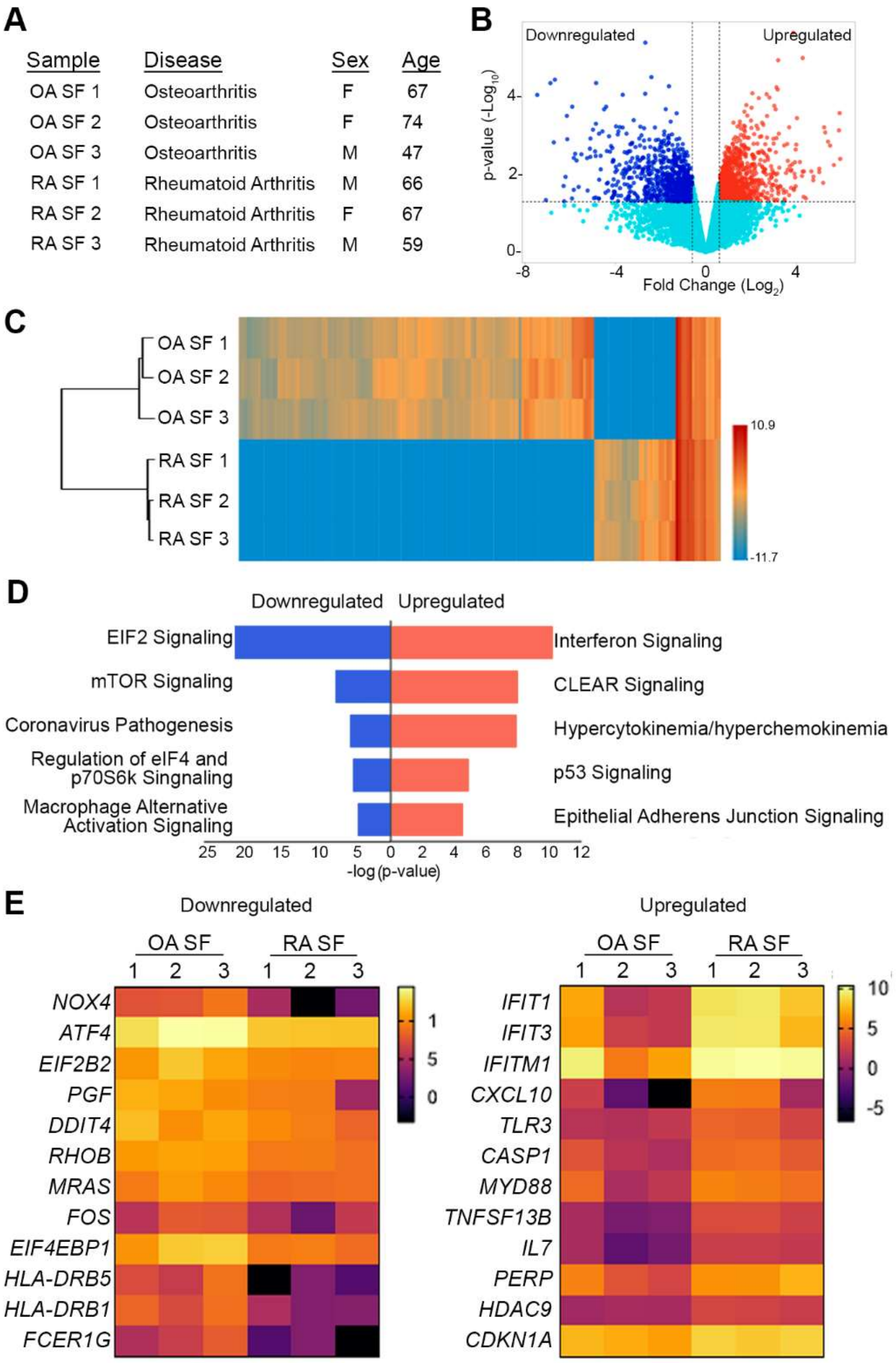

Fig. S2. Transcriptomic analysis revealed the upregulation of critical inflammatory mediators in RA SFs.

(A) Description of OA and RA SFs used in RNA-seq experiments. (B) Volcano plot of up and downregulated genes in RA SFs compared OA SFs by 1.5 FC and p-value < 0.05 (C) Hierarchical clustering of the genes differentially expressed genes by Euclidean distance and centroid linkage method (D). Ingenuity Pathway Analysis (Qiagen) showing the top 5 canonical pathways predicted to be associated with the genes up and downregulated in RA SFs. (E) Heatmaps Log2 RPKM values of genes associated with canonical pathways identified in panel D.

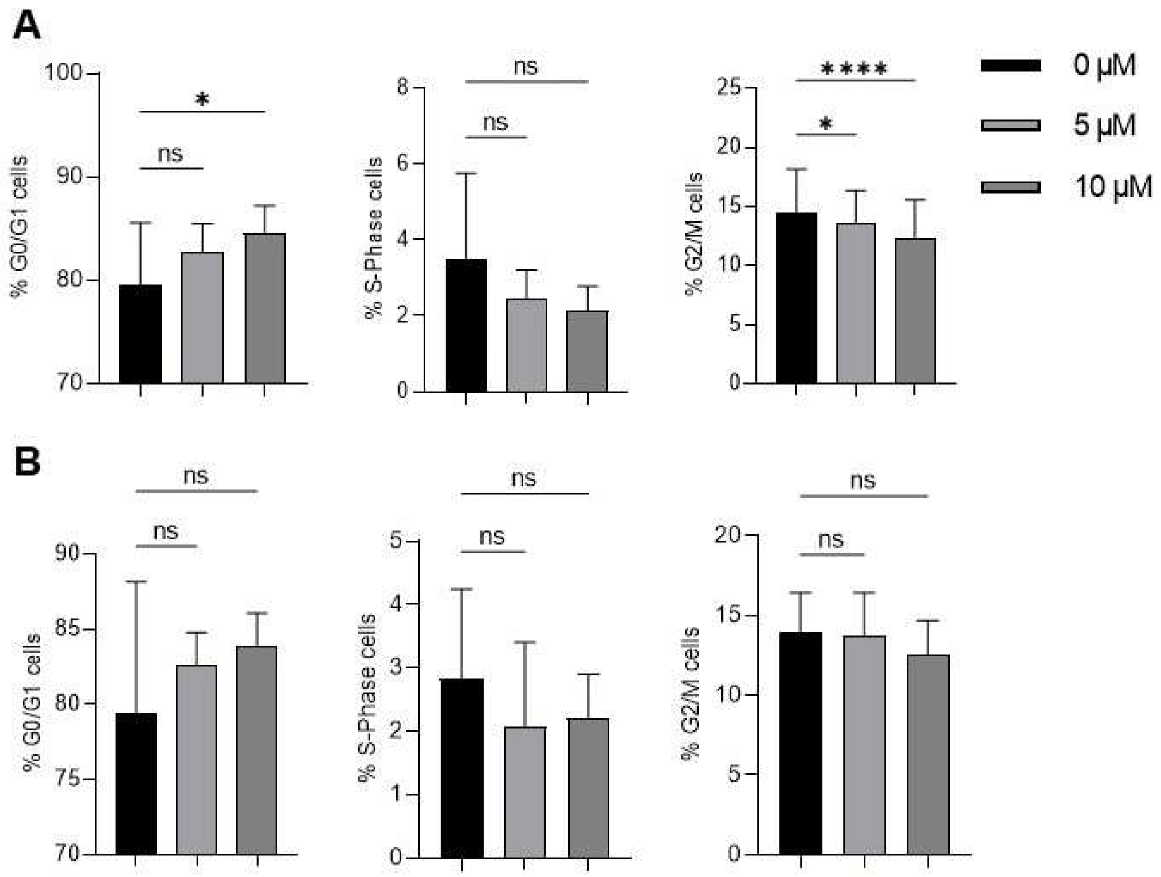

Fig. S3. Cell cycle analysis of RA-FLS treated with ST2825 at 48 and 72 h.

Quantification of the percentage of RA SFs in various phases of the cell cycle upon ST2825 treatment at 48 (A) and 72 (B) h by imaging cytometry. One-way ANOVA and Dunnett's multiple comparisons test were performed to determine statistical significance. *p<0.05, ****p<0.0001.

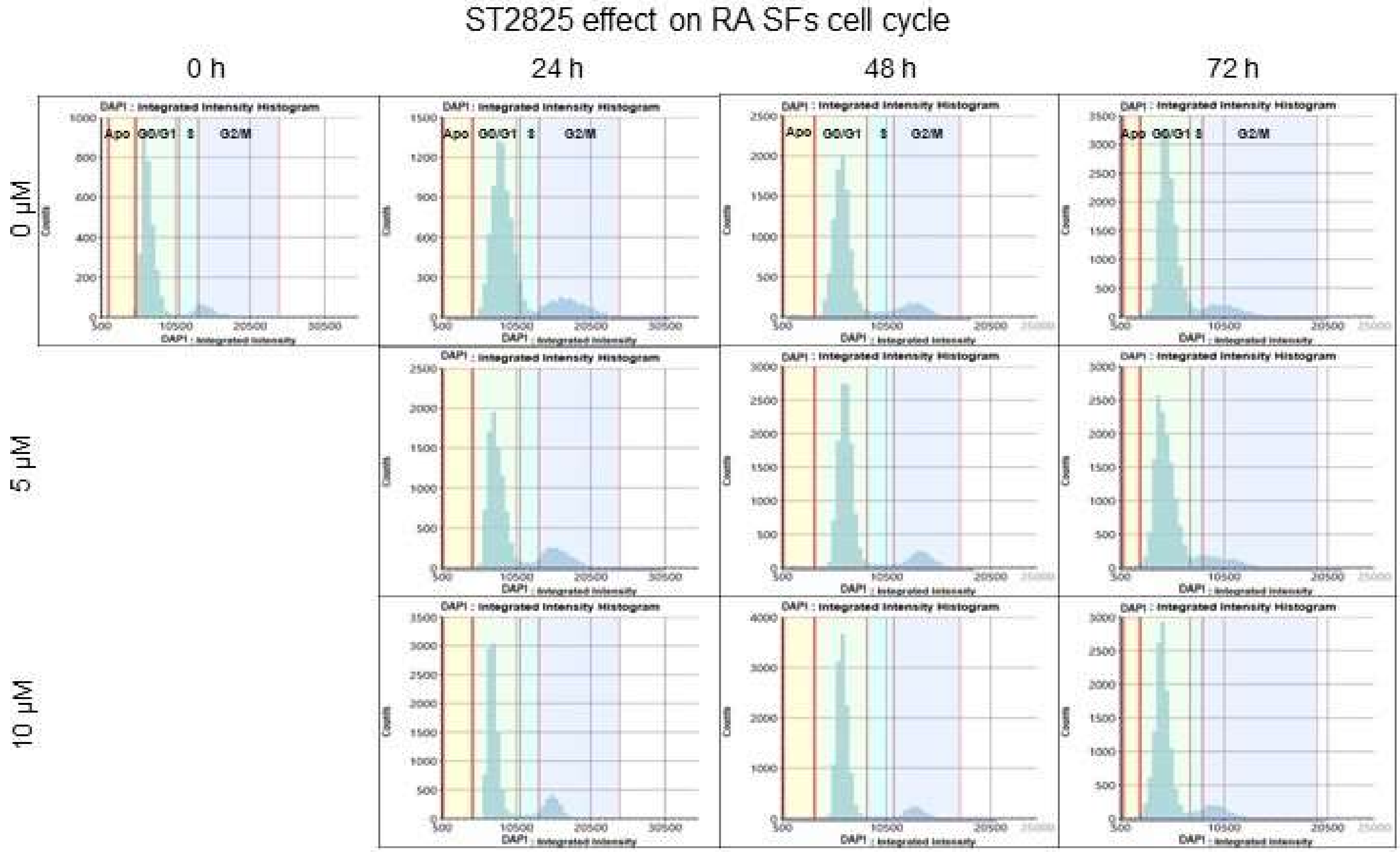

Fig. S4. Cell cycle analysis on RA SFs treated with ST2825.

Representative histograms show cell cycle differences among different ST2825 concentrations and time points. Apoptotic cells (yellow), G0/G1-phase cells (green), S-phase cells (blue), and G2/M-phase cells (purple) are shown in the figure. The y axes represent the total count of cells, and the x axes represents integrated intensity of DAPI measured by whole-well image cytometry.

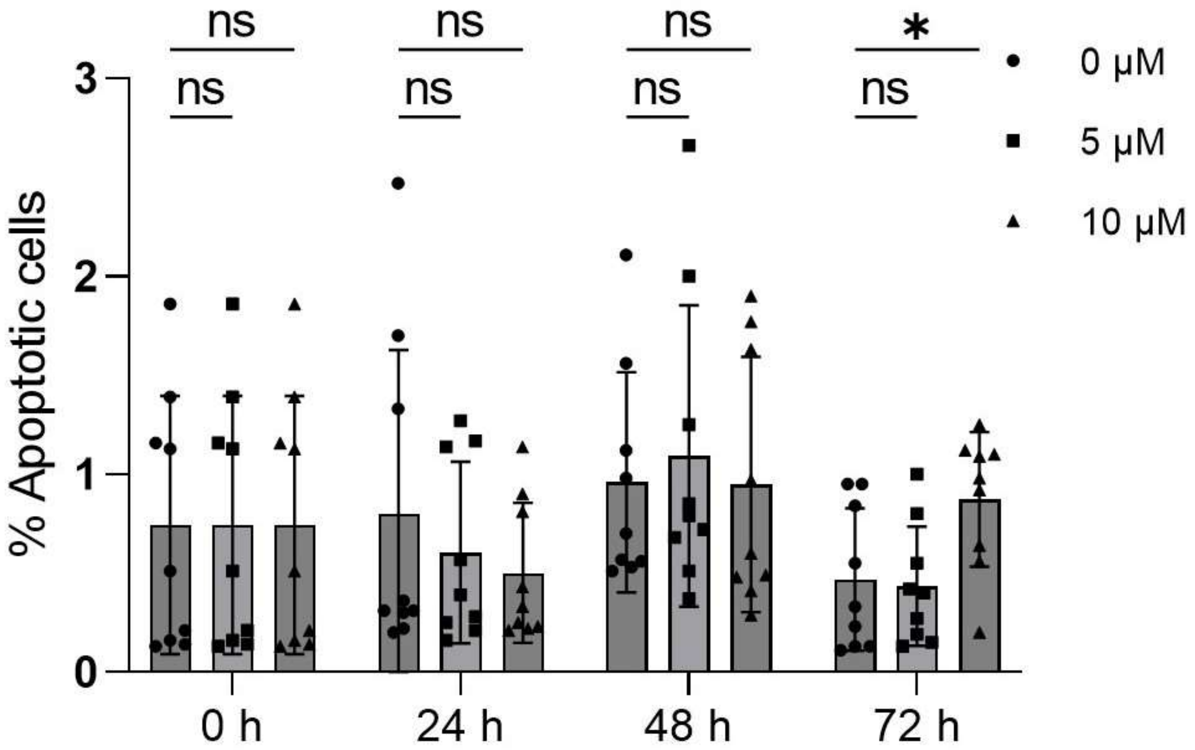

Fig. S5. Apoptosis analysis of RA-FLS treated with ST2825.

Apoptosis was determined on RA SFs treated with 0, 5, and 10 μM of ST2825 at 0, 24, 48, and 72 h. No statistically significant differences were observed after 24 and 48 h of incubation with ST2825. The percentage of apoptotic cells significantly increased after 72 h of incubation with 10 μM of ST2825 (*p*=0.0476). Two-way ANOVA and Dunnett's multiple comparisons test were used to determine statistical significane. **p*<0.05.

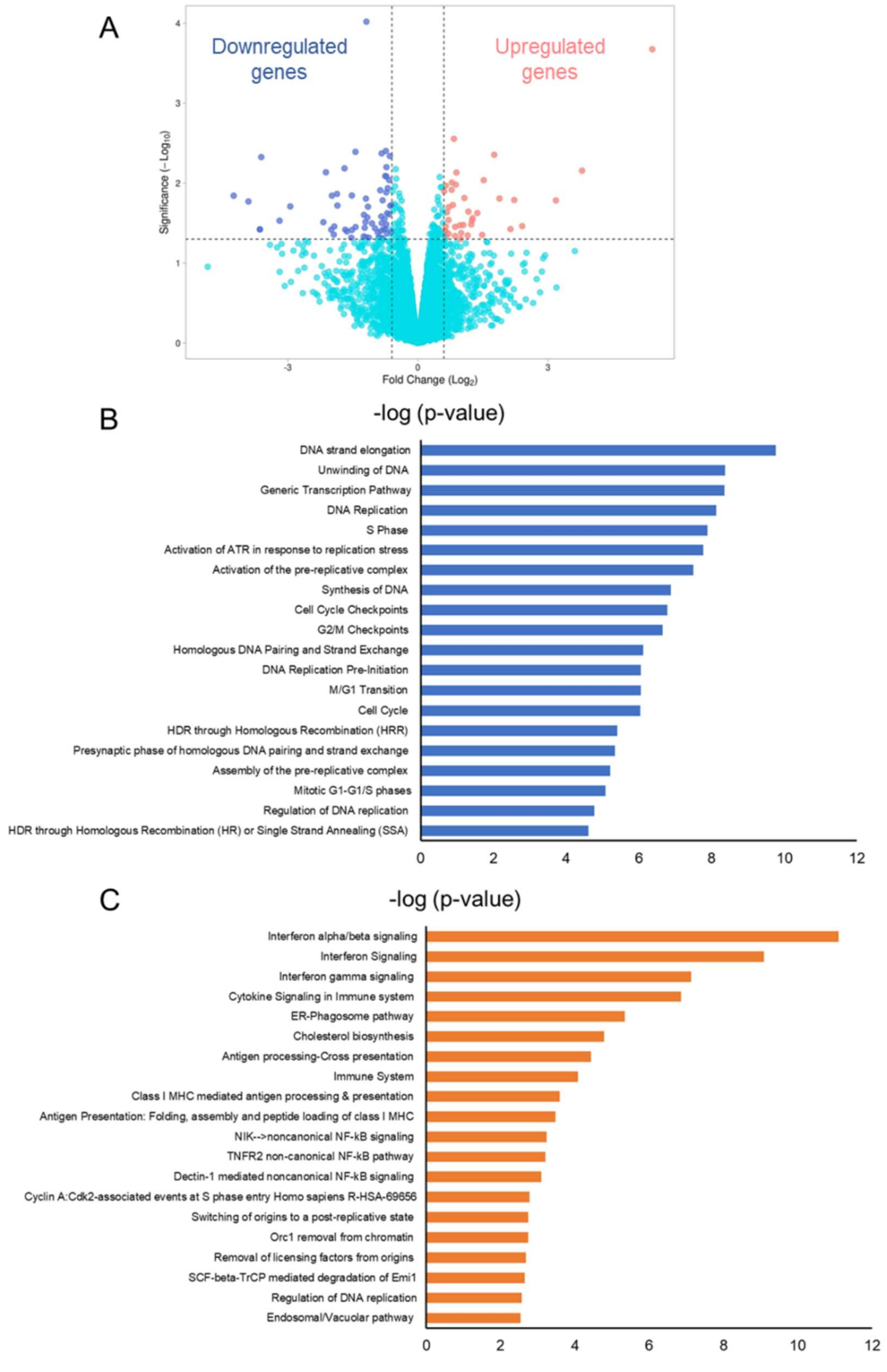

Fig. S6. Transcriptomic analysis identified DEGs and canonical pathways in RA SFs treated with ST2825.

(A) Volcano plot of up and downregulated genes in RA SFs compared ST2825-treated RA SFs by 1.5 FC and *p*-value < 0.05. Enriched pathway analysis showing the top 10 canonical downregulated (B) and upregulated (C) pathways predicted to be associated with the genes up and downregulated in RA SFs treated with ST2825.

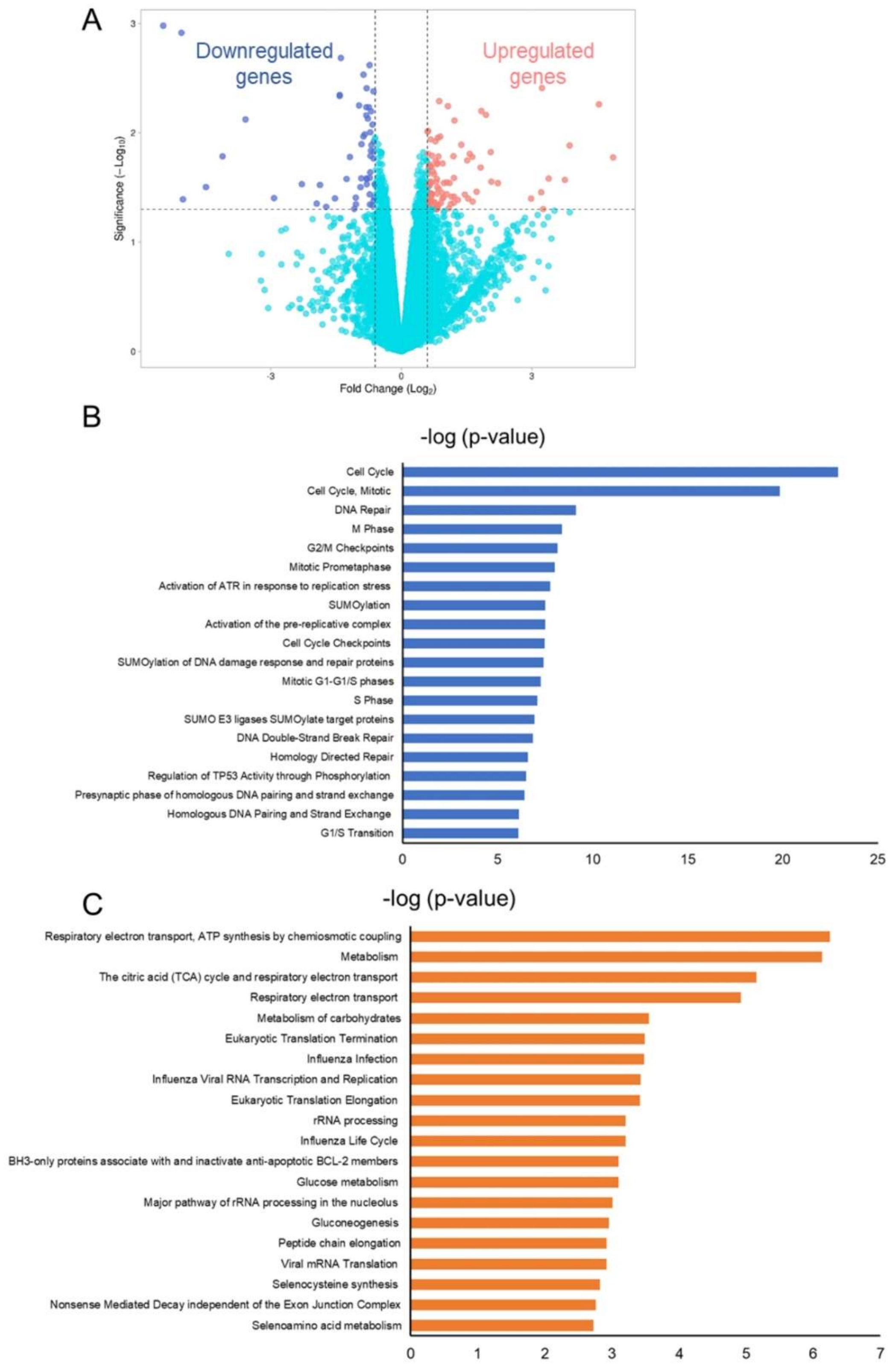

Fig. S7. Transcriptomic analysis identified the effect of ST2825 on DEGs and canonical pathways in LPS-treated RA SFs.

(A) Volcano plot of up and downregulated genes in LPS-stimulated RA SFs after treatment with ST2825 by 1.5 FC and *p*-value < 0.05. Enriched pathway analysis showing the top 10 canonical downregulated (B) and upregulated (C) pathways predicted to be associated with the genes up and downregulated in RA SFs.

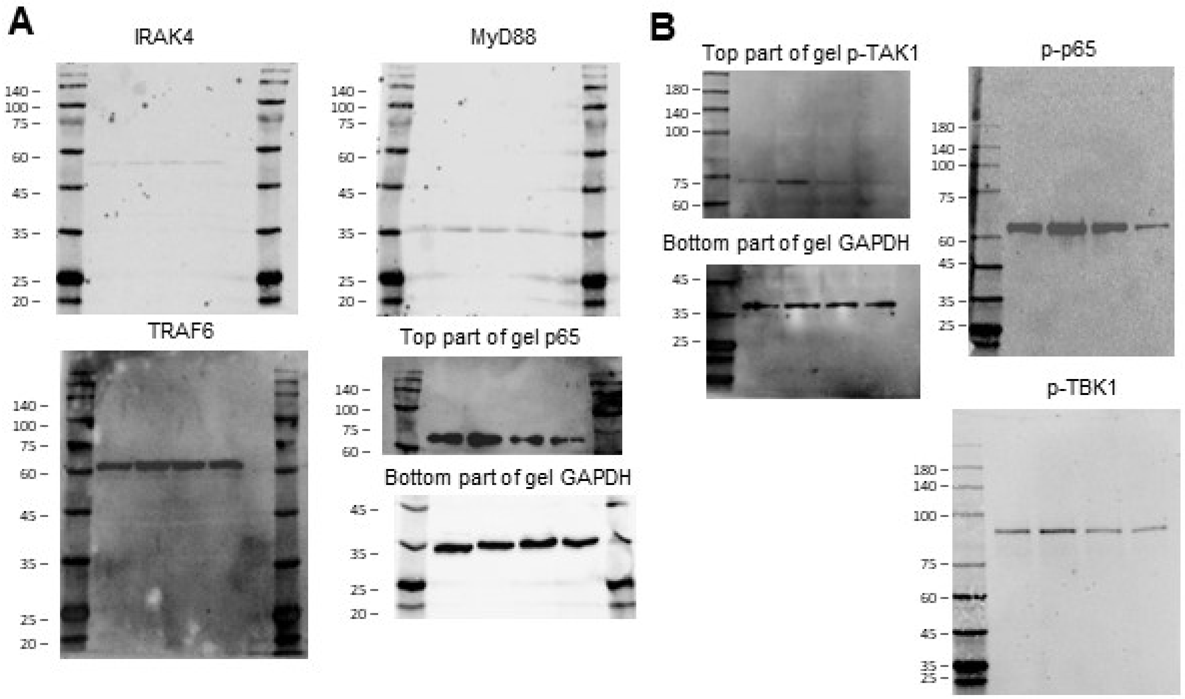

Fig S8. Raw data of western blot images. (A) Fig 5A and (B) Fig 5C.

Supplementary Table 1. Primer sequences for qRT-PCR.

Gene	Primer Sequences (5’ to 3’)	Exon Location	RefSeqNumber
TBP	F: GCTGTTTAACTTCGCTTCCG	1 - 2	NM_003194
	R: CAGCAACTTCCTCAATTCCTTG		
CCNE2	F: GCTTCAACTCATTGGAATTACCTC	7 - 8	NM_057749
	R: AGCACCATCAGTGACGTAAG		
MYBL2	F: GATTCCTGTAACAGCCTCACG	8 - 9	NM_002466
	R: CTCTCCAGCTCCAATGTGTC		
IL1B	F: TGTACGATCACTGAACTGCAC	5 – 6	NM_000576
	R: AAAGGACATGGAGAACACCA		
MYD88	F: CTGTGTCTGGTCTATTGCTAGTG	3a - 5	NM_003468
	R: TCTGATGGGCACCTGGA		

Supplementary Table 2. Antibodies used for western blot.

Name	*Company*	*Catalog*	*Dilution*
RELA-HRP	*Cell Signaling Technology*	*59674S*	*1:1000*
GAPDH-HRP	*Sigma-Aldrich*	*G9295-200UL*	*1:10000*
IRAK4	*Cell Signaling Technology*	*39433T*	*1:1000*
MyD88	*Cell Signaling Technology*	*39433T*	*1:1000*
p-p65	*Cell Signaling Technology*	*3033S*	*1:500*
TRAF6	*Cell Signaling Technology*	*39433T*	*1:1000*
p-TAK1	*Cell Signaling Technology*	*4536S*	*1:500*
p-TBK1	*Cell Signaling Technology*	*39433T*	*1:500*
